# Direct observation by time-resolved infrared spectroscopy of the bright and the dark excited states of the [Ru(phen)_2_(dppz)]^2+^ light-switch compound in solution and when bound to DNA†
†Electronic supplementary information (ESI) available: Experimental details, XRD spectra, spectra (UV-Vis, emission, IR and TRIR), tables of vibration, animations of vibrational modes of the complex and DFT calculation data. CCDC 1038710. For ESI and crystallographic data in CIF or other electronic format see DOI: 10.1039/c5sc04514b


**DOI:** 10.1039/c5sc04514b

**Published:** 2016-01-27

**Authors:** Fergus E. Poynton, James P. Hall, Páraic M. Keane, Christine Schwarz, Igor V. Sazanovich, Michael Towrie, Thorfinnur Gunnlaugsson, Christine J. Cardin, David J. Cardin, Susan J. Quinn, Conor Long, John M. Kelly

**Affiliations:** a School of Chemistry , Trinity College Dublin , The University of Dublin , Dublin 2 , Ireland . Email: jmkelly@tcd.ie; b Trinity Biomedical Sciences Institute (TBSI) , Trinity College Dublin , The University of Dublin , Dublin 2 , Ireland; c Department of Chemistry , University of Reading , Reading RG6 6AD , UK; d Diamond Light Source , Harwell Science and Innovation Campus , Didcot , Oxfordshire OX11 0QX , UK; e Central Laser Facility , Research Complex at Harwell , STFC Rutherford Appleton Laboratory , Oxfordshire OX11 0QX , UK; f School of Chemistry , University College Dublin , Dublin 4 , Ireland; g The School of Chemical Sciences , Dublin City University , Dublin 9 , Ireland . Email: conor.long@dcu.ie

## Abstract

The [Ru(phen)_2_(dppz)]^2+^ complex (**1**) is non-emissive in water but is highly luminescent in organic solvents or when bound to DNA, making it a useful probe for DNA binding. To date, a complete mechanistic explanation for this “light-switch” effect is still lacking. With this in mind we have undertaken an ultrafast time resolved infrared (TRIR) study of **1** and directly observe marker bands between 1280–1450 cm^–1^, which characterise both the emissive “bright” and the non-emissive “dark” excited states of the complex, in CD_3_CN and D_2_O respectively. These characteristic spectral features are present in the [Ru(dppz)_3_]^2+^ solvent light-switch complex but absent in [Ru(phen)_3_]^2+^, which is luminescent in both solvents. DFT calculations show that the vibrational modes responsible for these characteristic bands are predominantly localised on the dppz ligand. Moreover, they reveal that certain vibrational modes of the “dark” excited state couple with vibrational modes of two coordinating water molecules, and through these to the bulk solvent, thus providing a new insight into the mechanism of the light-switch effect. We also demonstrate that the marker bands for the “bright” state are observed for both Λ- and Δ-enantiomers of **1** when bound to DNA and that photo-excitation of the complex induces perturbation of the guanine and cytosine carbonyl bands. This perturbation is shown to be stronger for the Λ-enantiomer, demonstrating the different binding site properties of the two enantiomers and the ability of this technique to determine the identity and nature of the binding site of such intercalators.

## Abstract 2

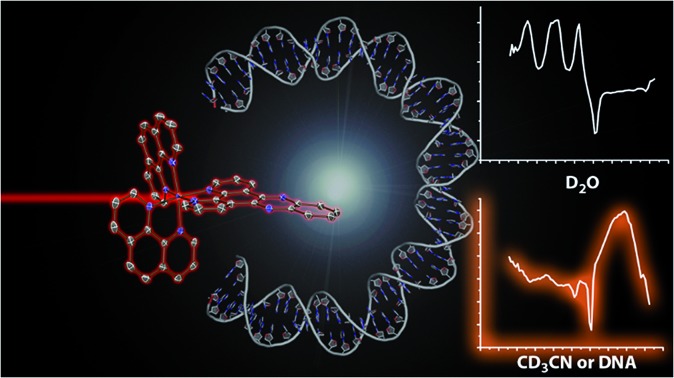
Strikingly different TRIR spectra are recorded for the complex in D_2_O or CD_3_CN or when DNA-bound.

## Introduction

**Fig. 1:**
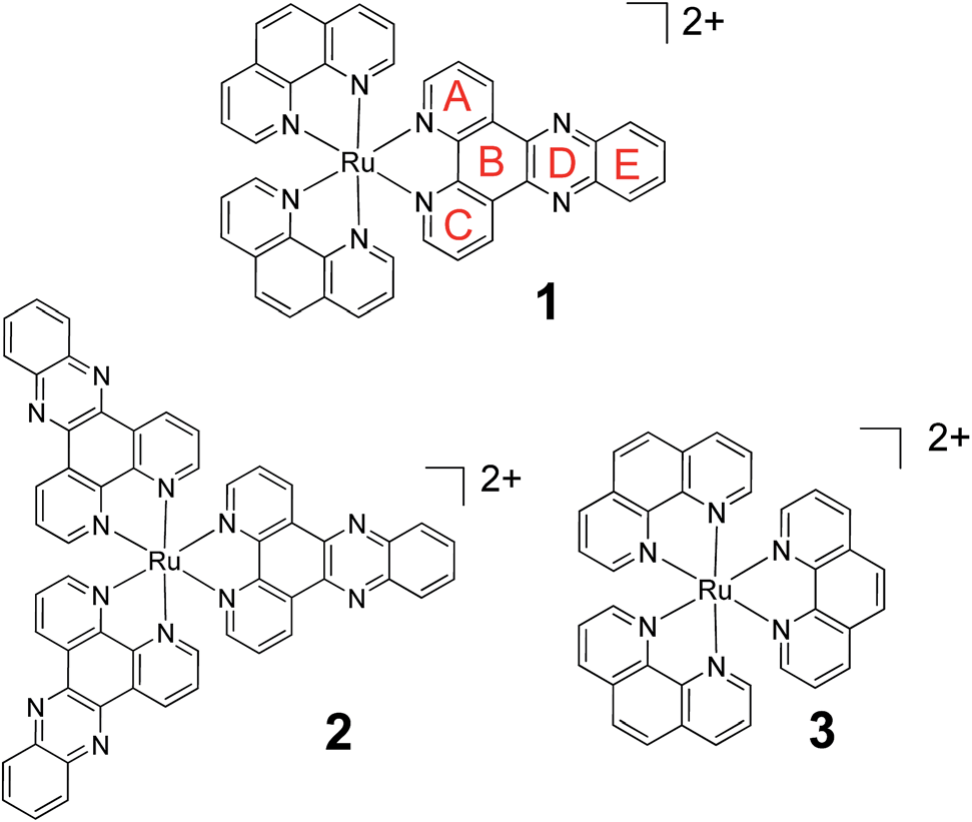
The chemical structure of **1**, **2** and **3** showing ring lettering codes used to describe the dppz ligand.

Molecular probes, whose luminescence is “switched on” in the presence of an analyte are attractive to researchers as both diagnostic and imaging agents due to the extremely low limit of detection achievable with such systems. However, in order to design and optimise new probes, a detailed understanding of the photophysical processes responsible for this “switch on” in luminescence is essential. One particular class of ruthenium polypyridyl complexes, based on the dppz ligand (dppz = dipyrido[3,2-*a*:2′,3′-*c*]phenazine), has been the focus of significant investigation since the first reports of their remarkable luminescence behaviour.^1^,^2^ [Ru(phen)_2_(dppz)]^2+^ (phen = 1,10-phenanthroline) (**1**) (Fig. 1), for example, is only very weakly emitting in water but is strongly luminescent in non-protic organic solvents or when bound to double-stranded DNA.^3^–^6^ This makes **1** valuable as a “light-switch” for the detection of double-stranded DNA,^2^ and consequently an excellent candidate for luminescence imaging and biological profiling within cancer cells.^7^–^14^ Furthermore, metal complexes of this type may also have potential as photo-therapeutic agents.^15^–^18^

In an effort to understand the origin of this light-switch effect, the photophysical and excited-state properties of Ru-dppz complexes have been the focus of numerous studies.^3^–^6^,^19^–^42^ Earlier photophysical studies were consistent with the luminescence originating from a triplet metal-to-ligand charge-transfer (^3^MLCT) excited state with increased electron density localised on rings A–C of the dppz ligand (Fig. 1). By contrast it was proposed that in the non-luminescent “dark” state (*i.e.* in water), the increased electron density is concentrated on the phenazine portion of the ligand (*i.e.* rings B, D, E).^19^–^30^ Binding of water molecules onto the nitrogens of ring D was considered to be a key factor in the non-radiative deactivation of the “dark” excited state, although the nature of interaction with the water molecules has not been fully established.

Many investigations into the nature of the light-switch behaviour of **1** and related compounds have relied on standard luminescence techniques.^23^–^30^ However, such methods only provide information on the luminescent “bright” excited state species. Hence the use of other spectroscopic techniques that can yield information on both the “bright” and the non-emissive “dark” states needs to be considered. Previous studies have used transient visible absorption (TA)^31^,^32^ or time-resolved resonance Raman (TR^3^) spectroscopy for this purpose.^33^–^42^ Time-resolved techniques reveal the excited state lifetime of **1** in aerated CH_3_CN to be 180 ns but only 250 ps in H_2_O.^5^,^31^ While these studies clearly point to the presence of two distinct excited states, there has been some disagreement about the nature of these states in the literature, especially from computational studies.^19^–^22^

Time-resolved infrared (TRIR) spectroscopy can provide detailed structural information about excited states as well as providing excellent kinetic data.^43^–^50^ This technique has the advantage of probing the specific vibrations of the polypyridyl ligands, being sensitive to changes in electronic structure and has spectral resolution superior to that of the TR^3^ technique.^51^ However, to the best of our knowledge there has been no study of the excited states of **1** using TRIR. Such a study is reported below, which includes the homoleptic complexes [Ru(dppz)_3_]^2+^ (**2**) and [Ru(phen)_3_]^2+^ (**3**) in both CD_3_CN and D_2_O, where we show for the first time that the “bright” and “dark” excited states of **1** have strikingly different TRIR spectra. This has allowed marker bands to be identified for **1**, which enables the excited state species produced in a particular medium to be readily identified. Comparative studies with **2** and **3** reveal that these marker bands correspond to vibrations associated with the dppz ligand. The nature of these vibrational modes was further characterised using DFT calculations, and in D_2_O numerous modes of vibration show strong coupling to the hydrogen bonded water molecules. Finally we demonstrate that these spectral signatures can be used to identify the “bright” excited state species of **1** when bound to DNA.

## Results and discussion

### Characterisation of complexes **1**, **2** and **3**

**Fig. 2:**
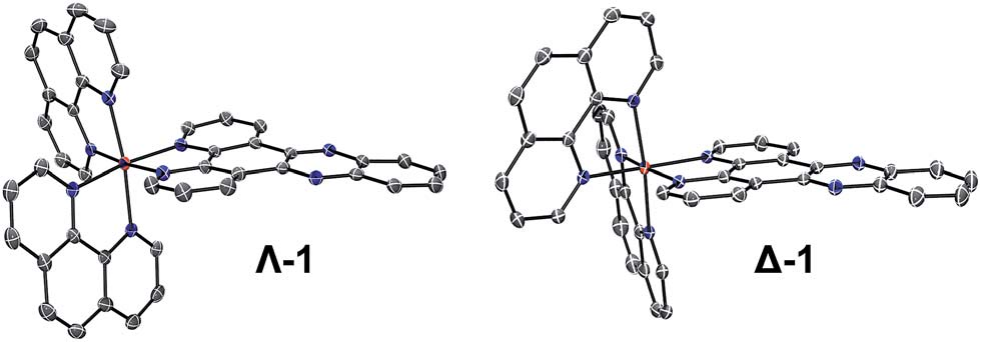
The molecular structure of Λ- and Δ-**1** obtained by single crystal X-ray diffraction (ESI†) (PF_6_^–^ counterions and solvent molecules are omitted for clarity – see the ESI† for full structure; ellipsoids are shown at 50% probability).

Complexes **1**, **2** and **3** were synthesised as their Cl and PF_6_ salts by modified literature procedures (see ESI† for details).^6^,^16^,^52^ Red needle-shaped crystals of [**1**] 2PF_6_ were grown by vapour diffusion of diethyl ether into a racemic solution of the complex in CH_3_CN. The molecular X-ray structures of Λ- and Δ-**1** are shown in Fig. 2. The asymmetric unit contains two complexes (one Λ and one Δ-enantiomer) and four PF_6_^–^ counterions. The complexes exhibit distorted octahedral geometries around the metal atom, with Ru–N bond distances between 2.058(3)–2.078(2) Å and the dppz ligand exhibits a slight distortion from planarity. These observations are similar to those found in the crystal structure of [Ru(bpy)_2_(dppz)]^2+^ (bpy = 2,2′-bipyridine) (see Fig. S1–S4 and Tables S1 and S2 in the ESI† for further details).^53^,^54^

**Fig. 3:**
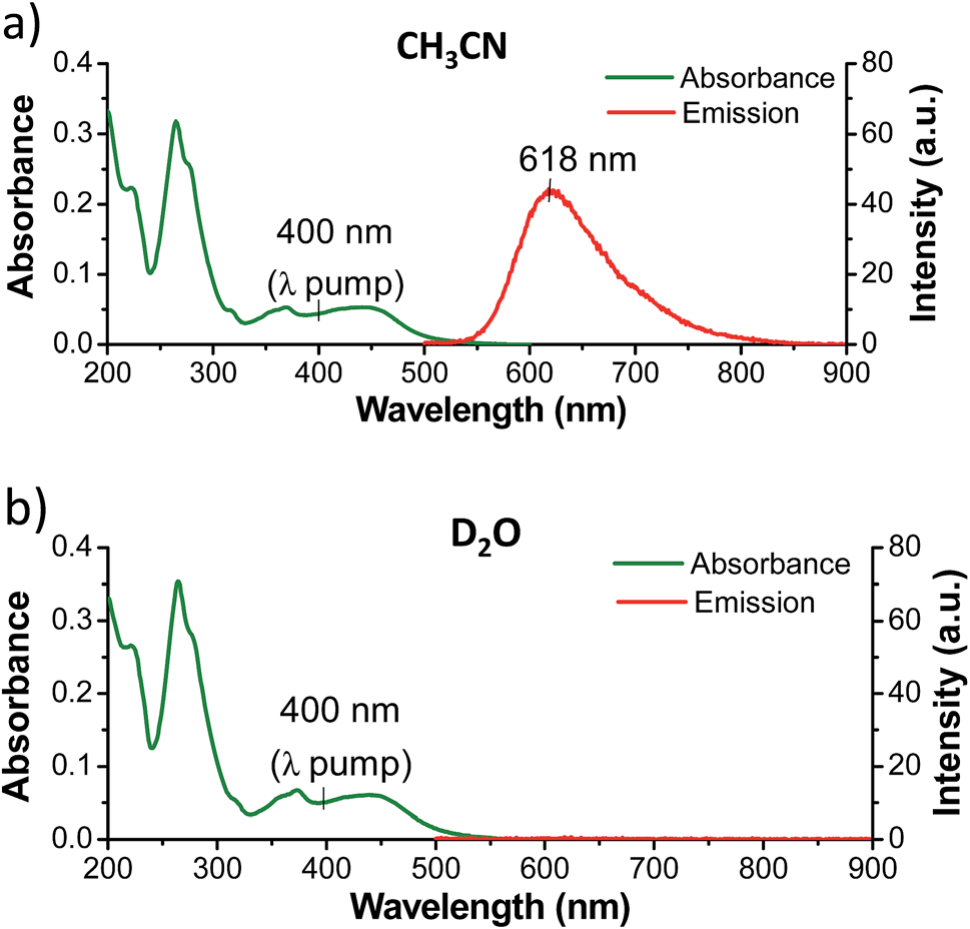
UV/Vis absorption and emission spectra (*λ*_ex_ = 410 nm) of **1** (3.0 µM) in (a) CH_3_CN and (b) H_2_O, showing the solvent dependent luminescence of **1**. The excitation wavelength for the TRIR experiments is also highlighted.

**Fig. 4:**
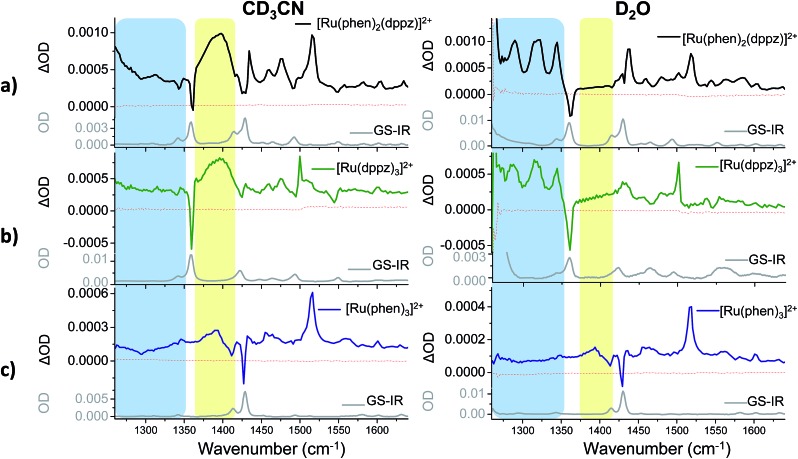
Time-resolved infrared (TRIR) spectra recorded 35 ps after 400 nm excitation of **1**·2PF_6_^–^ (500 µM), **2**·2PF_6_^–^ (500 µM) and **3**·2Cl^–^ (500 µM) in CD_3_CN (left column) and of **1**·2Cl (500 µM), **2**·2Cl (300 µM) and **3**·2Cl (500 µM) in D_2_O (right column) and their corresponding ground state FTIR (GS-IR) spectra. The blue and yellow coloured regions highlight the characteristic transient bands for the “bright” and “dark” excited states. (a–c) refer to the TRIR spectra of complex **1**, **2** and **3** in both solvents.

While complexes **1**, **2**, and **3** are emissive in CH_3_CN solution, no luminescence is detectable for the dppz-containing complexes **1** and **2** in water under comparable experimental conditions (Fig. 3 and S5 in the ESI†).^6^,^55^ In contrast **3** was found to be emissive in water (Fig. S6 in the ESI†). The ground state infrared spectrum of each complex was also recorded in both CD_3_CN and D_2_O solution (Fig. 4 (GS-IR)). These IR spectra clearly show distinct vibrations for the dppz (1325–1375 cm^–1^) and phen (1400–1450 cm^–1^) ligands. Moreover these measurements do not reveal any significant solvent effects on the position or intensity of the vibrational bands.

### Time-resolved infrared studies

TRIR difference spectra of complexes **1–3**, which were recorded in the mid infrared (1260–1650 cm^–1^) on the picosecond timescale after excitation with a 50 fs 400 nm pulse, are shown in Fig. 4. These present the IR spectrum of the excited state minus that of the ground state and this spectral region probes the aromatic ring breathing modes of the complexes.^56^ The spectra shown in Fig. 4 were recorded after 35 ps as at earlier times (less than 20 ps) they are expected to be influenced by vibrational relaxation and other inter-state processes.^31^–^42^

The bands of the ground state appear as negative ‘bleach’ peaks (for **1** in CD_3_CN these are found at 1361, 1430 and 1492 cm^–1^). New transient (positive) absorption bands, arising from vibrations of the excited state, are also observed. For **1** in CD_3_CN a strong and broad band at 1396 cm^–1^ is evident (shown within the yellow box in Fig. 4a). By contrast the TRIR spectrum of **1** recorded in D_2_O shows three prominent sharp transient bands at 1290, 1321 and 1345 cm^–1^ (blue box in Fig. 4a), while the strong broad transient at 1396 cm^–1^ found in CD_3_CN is absent. In both solvents a strong sharp transient band at 1518 cm^–1^ is present.

To aid in the assignment of the key features observed in the TRIR spectra of **1**, analogous spectra of the homoleptic complexes **2** and **3** were also recorded (Fig. 4b and c). As mentioned above, the tris(dppz) complex **2** exhibits similar solvent dependent luminescence behaviour to **1**,^55^ whereas the tris(phen) complex **3** is luminescent in both organic and aqueous solution. The TRIR spectra of **2** contain a distinctive broad transient band at 1396 cm^–1^ in CD_3_CN and three sharp transient bands at 1290, 1321 and 1345 cm^–1^ in D_2_O, behaviour similar to that observed in the TRIR of **1**. For **3** the TRIR spectra are closely similar in both solvents. This is expected as **3** does not show a light-switch effect. The sharp bands found in D_2_O for **1** and **2** are notably absent for **3**, and while **3** in CD_3_CN does show a broad band at about 1390 cm^–1^, it is markedly lower in intensity than is found for **1** or **2**. These findings strongly suggest that the bands in **1** are associated with the dppz ligand and may be used as distinct marker bands for the “bright” and “dark” excited states of **1**. A strong transient band at 1500 cm^–1^ is observed for **2** in both CD_3_CN and D_2_O, but in the TRIR spectra of **1** the intensity of the equivalent band is greatly reduced and it is only observed as a shoulder in CD_3_CN and as a very weak band in D_2_O. The sharp transient band at 1518 cm^–1^ seen in the TRIR spectra of **1** in both solvents is also present in the spectrum of the tris(phen) complex **3**, but is absent from that of **2**. This suggests that this vibration originates from the ancillary phen ligands. This band, as well as the other weak transient bands seen in the TRIR spectrum of **3** are consistent with those previously reported by step-scan time-resolved IR measurement, although their relative intensities are different.^57^‡
‡In the current study, the TRIR spectral features of the tris(phen) complex **3** were the same irrespective of excitation wavelength (*λ*_ex_ = 400 or 355 nm) or time (between 35 ps and 1000 ns) (Fig. S7 and S8 in the ESI†). This indicates that the differences observed are not due to different electronic excited states of **3** in the two studies. It may be noted that the concentration of **3** is significantly lower in the current study. This might be a reason for the discrepancy. However, this variation could also arise due to the different experimental techniques used in the two studies.


**Fig. 5:**
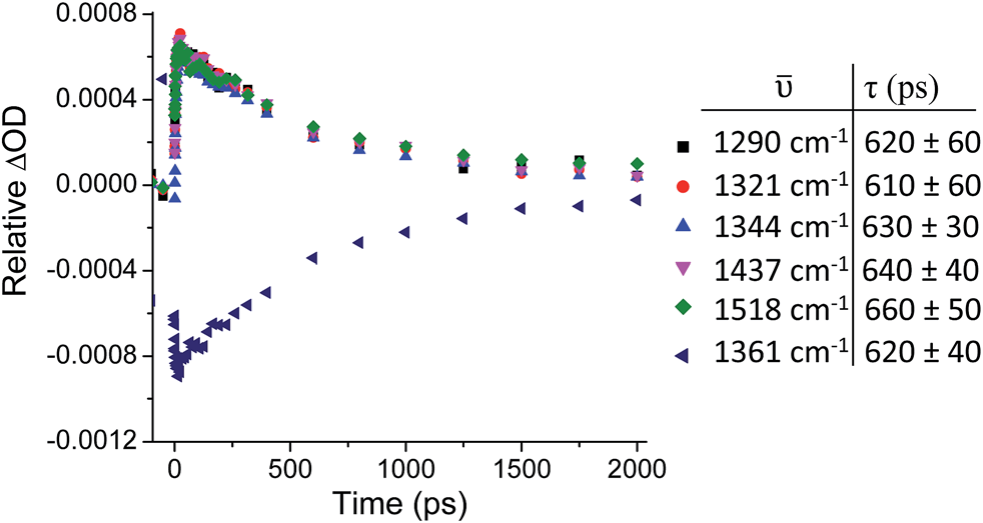
ps-TRIR (*λ*_ex_ = 400 nm) decay kinetics of the transient bands at 1290, 1321, 1344, 1437 and 1518 cm^–1^ and bleach at 1361 cm^–1^ of **1**·2Cl (500 µM) in D_2_O.

Kinetic analysis of the five major transient bands in the TRIR spectrum of **1** in D_2_O, as well as the bleach at 1361 cm^–1^, yielded an excited-state lifetime of 630 (±30) ps (see Fig. 5). This confirms that all bands in the TRIR spectrum originate from a single species in solution, which has a similar lifetime to that previously determined by transient absorption measurements, where it was also shown that the lifetime in D_2_O is more than twice that in H_2_O.^31^ The lifetime of the excited state of **1** in CD_3_CN was too long to be measured using the current ps-TRIR experimental set-up. The lifetime of **2** in D_2_O was determined to be 1400 ps.

### Computational analysis

The differences in the TRIR spectra of **1** indicate that there must be significant and distinct changes to the electron distribution in the dppz ligand of the excited states in the two solvents. To explore the origin of these changes in the TRIR spectrum the spectra were simulated using computational methods. Previous researchers have proposed that the “dark” state in aqueous solution is one in which water molecules bind to the N-atoms of the phenazine (ring D), producing an excited state which rapidly undergoes non-radiative deactivation.^3^,^23^–^42^ To determine whether such species would have the characteristic spectra observed in the above TRIR experiments, the lowest energy triplet states of **1** and **1**·2(D_2_O) were modelled using a hybrid density functional method (B3LYP for singlet species and UB3LYP for triplet)^58^–^61^ and a double zeta quality basis set§
§Similar results were obtained with the triple zeta potential; however, due to computational cost IR spectra had to be calculated using the double zeta potential model. (LanL2DZ)^62^–^66^ as implemented in the Gaussian 09 (Revision D.01) program suite. Corrections for solvents were applied using the Polarisable Continuum Model (PCM).^67^ To simulate the IR spectra measured in D_2_O, calculations were performed using the PCM model for bulk water but with the complex **1**·2(D_2_O), where two D_2_O molecules are hydrogen-bonded to the phenazine N atoms (ring D) of the dppz ligand (full details of these calculations are presented in the ESI†).

**Fig. 6:**
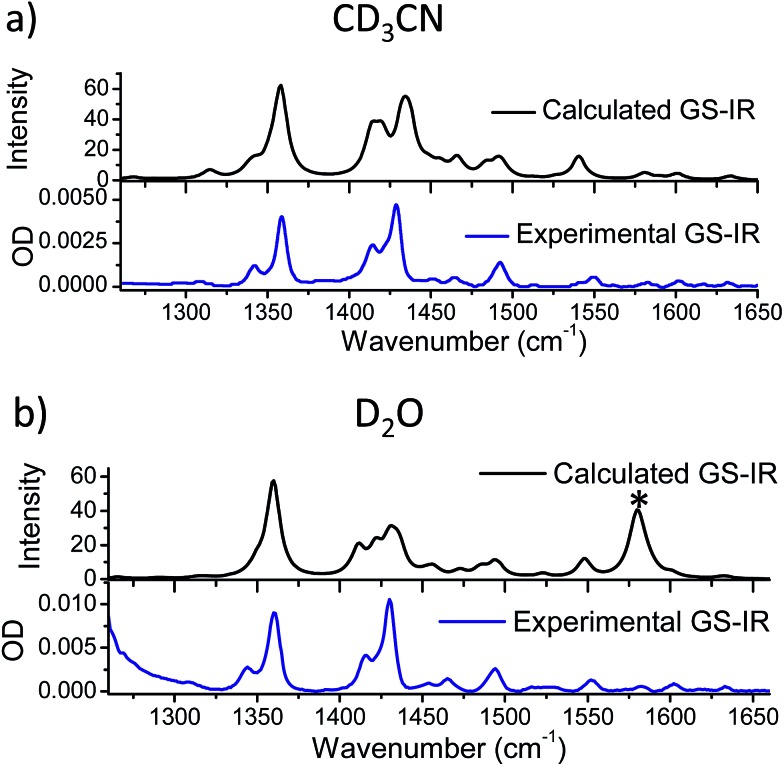
Comparison of the calculated ground state IR spectrum of 1 in (a) acetonitrile and (b) water with the FTIR spectrum of the complex in CD_3_CN and D_2_O at 1 mM, respectively (* see footnote¶
¶The calculated band at 1580 cm^–1^ in D_2_O is composed of a number of O–D–O bending modes. In these calculations the bulk water was not modelled and therefore differences between the calculated and experimental O–D vibrations are to be expected.).

**Fig. 7:**
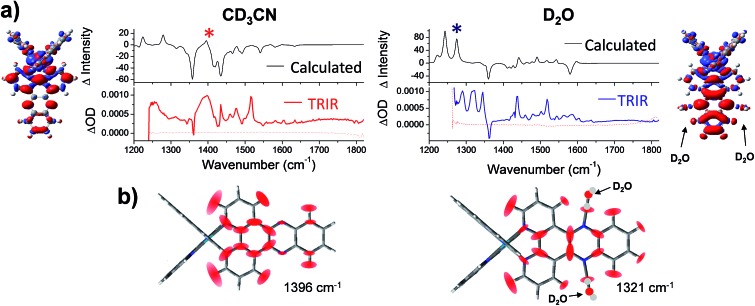
(a) Simulated difference spectra compared with the experimental TRIR spectra of **1** in acetonitrile (left column); and **1**·2(D_2_O) in water (right column). In the experimental spectra, wavenumbers below 1250 cm^–1^ were inaccessible due to strong absorbance of both solvents in this region. Structural inserts show electron density difference map (iso level 0.0008) between the lowest energy triplet state of 1 minus the electron density of the equivalent singlet ground-state at the same geometry in acetonitrile (left hand side) and for **1**·2(D_2_O) in water (right hand side) (the red and blue volumes indicate the regions where the electron density is respectively higher and lower in the triplet state compared to the ground state); (b) graphical illustration of the in-plane vibrational modes of the marker bands of the “bright” and “dark”: states of **1** at 1396 cm^–1^ in CD_3_CN and 1321 cm^–1^ in D_2_O (marked with asterisks in the spectra). Red ellipsoids indicate the regions of largest atomic displacement. Animations of these vibrations can be found online in the ESI† (Animation 1-CD_3_CN and Animation 2-D_2_O).

Using these methods the optimised geometry of the ground state of **1** was found to be in good agreement with the experimentally obtained values from X-ray diffraction studies (Table S6 in the ESI†). The infrared spectra of both the ground state in acetonitrile and water were simulated and found to reproduce well the principal features of the FTIR spectra, shown in Fig. 6, indicating that the calculations effectively modelled the ground electronic state of **1**. The calculated change in electron density distribution between the ground state and the lowest triplet state was found to be quite different between the species in acetonitrile and in water. In acetonitrile an increase in electron density was observed on the phenanthroline portion of the dppz (rings A–C), while in water, it increased more on the phenazine portion of the dppz ligand (rings C–E) (Fig. 7A), which is consistent with previous researchers.^29^,^34^

A decrease in electron density on the Ru metal centre was also observed in both cases, which indicated that the triplet lowest excited states in both solvents have substantial metal-to-ligand charge-transfer character, with additional small contributions from a purely dppz ligand-centred transition.^19^–^22^

Next a calculated IR difference spectrum in each solvent was generated by subtracting the calculated (singlet) ground state IR spectrum from the lowest energy triplet state. These difference spectra were found to be solvent dependent and contained the characteristic features of the experimental TRIR spectra (Fig. 7a and S11 in the ESI†). In particular, the broad band observed at 1396 cm^–1^ in CD_3_CN is observed in the simulated spectrum, while a structured spectrum at lower wavenumber is predicted in D_2_O. The intensity of the strong ancillary phen-based 1518 cm^–1^ transient band observed in the TRIR spectra of **1** in both solvents was found to be underestimated in the calculated IR difference spectra. However weak bands observed in the calculated IR spectra in both CD_3_CN and D_2_O were indeed found to correspond to vibrations localised on the ancillary phen ligands.

The nature of the major vibrational modes (at 1396 cm^–1^ in CD_3_CN and 1321 cm^–1^ in D_2_O) which represent characteristic “bright” and “dark” state marker bands are illustrated in Fig. 7b. Both of these modes were found to correspond to vibrations predominantly localised on the dppz ligand. Interestingly, in aqueous media the dppz-based mode of vibration at 1321 cm^–1^ was found to be coupled with the two hydrogen bonding D_2_O molecules (see Animation 2 in the ESI†). It could be envisaged that this strong coupling between vibrational modes of **1** and the D_2_O molecules could allow direct access to the vibrational states of the bulk solvent, thus providing the possible mechanism of the light-switch effect. By efficient transfer of excess energy to the bulk solvent, **1** rapidly returns back to the ground state (630 ps in D_2_O and 250 ps in H_2_O). This hypothesis was further investigated by modelling the complex *via* calculations with added H_2_O or D_2_O molecules, fourteen in total, two clusters of seven water molecules, one on each side of the dppz ligand. These clusters were constructed to model the behaviour of bulk water through hydrogen bonds with the phenazine nitrogen atoms. The clusters were limited to seven water molecules because as the cluster size increased the potential energy wells in which the water molecules sit on the hypersurface become more shallow, causing difficulty in optimising the geometry of the complex-cluster system. The remote water molecules in the clusters tend to exhibit small imaginary vibrations (negative eigenvalues of the Hessian matrix) reflecting the difficulty in locating the minimum energy structure. Notwithstanding these difficulties the eigenvalues of the Hessian matrix (constructed by the second derivative of the energy along all the vibrational degrees of freedom) which involve in-plane displacements of atoms in the dppz ligand also involve displacements of the atoms in the water clusters. This indicates that vibrations of the dppz ligand can couple with vibrations of remote water (either H_2_O or D_2_O) molecules in the clusters (see Animation 3-D_2_O cluster and Animation 4-H_2_O cluster in the ESI†).

### TRIR investigation with DNA

**Fig. 8:**
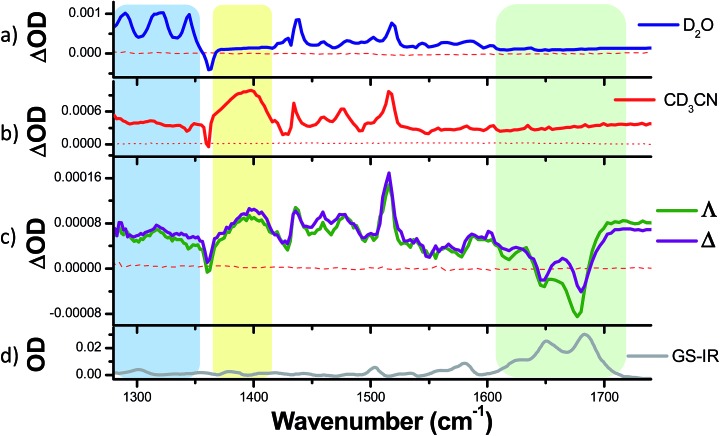
TRIR spectra recorded 35 ps after 400 nm excitation of (a) **1**·2Cl^–^ (500 µM) in D_2_O, (b) **1**·2PF_6_^–^ (500 µM) in CD_3_CN and (c) Δ- and Λ-[**1**] 2Cl^–^ (400 µM) and a duplex DNA oligonucleotide d(TCGGCGCCGA)_2_ (500 µM duplex) and (d) FTIR spectrum of oligonucleotide d(TCGGCGCCGA)_2_ (500 µM duplex) in deuterated potassium phosphate buffer (50 mM) pH 7. The blue and yellow coloured regions highlight the characteristic transient bands for the excited state of **1**, while the green coloured region highlights the bands associated with the DNA base pairs.

**Fig. 9:**
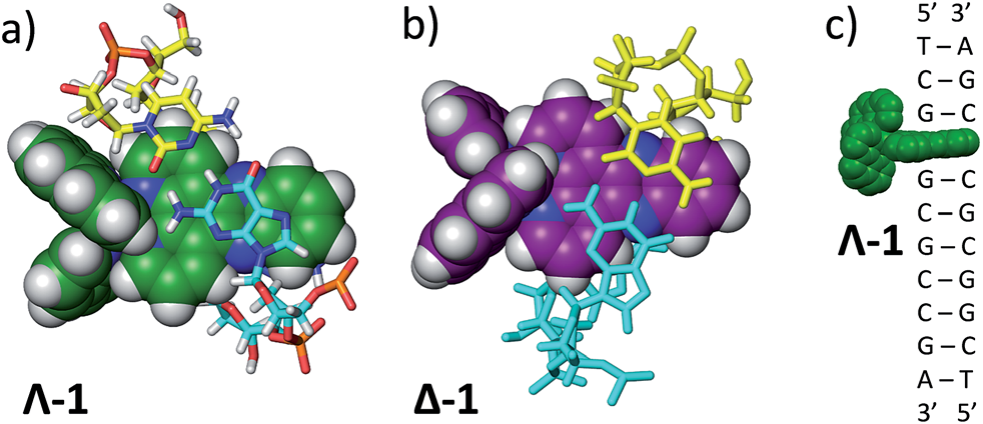
(a) Angled intercalation by Λ-**1** into the terminal CC/GG step in d(CCGGATCCGG).^71^ Note that the dppz is located under the purine (guanine) bases. Bases are coloured according to atom type with nitrogen in blue, oxygen in red, hydrogen in white and phosphorus in orange. Carbon atoms are cyan for guanine, yellow for cytosine and green for Λ-**1**; (b) a hypothetical model of an angled CC/GG intercalation site for Δ-**1**, constructed by direct manipulation of the crystal structure, to illustrate the difference in binding if the dppz group were directed further towards the pyrimidine bases (cyan). Purine bases are drawn in cyan; (c) schematic diagram of the binding of Λ-**1** at the G_3_G_4_/C_7_C_8_ step.

To ascertain whether these solvent results are pertinent to the behaviour in DNA the TRIR spectra of the Δ and Λ enantiomers of **1** bound to a DNA decamer duplex of sequence (TCGGCGCCGA)_2_ were also measured.^68^ Of particular interest were (i) if the marker bands of the “bright” state in CD_3_CN are also apparent in the “bright” state when bound to DNA and (ii) if there are differences between the TRIR spectra of the two enantiomers when bound to DNA. These TRIR spectra of **1** (Fig. 8c) show features similar to those found in CD_3_CN solution – particularly the broad transient band at 1396 cm^–1^ and the notable absence of the three sharp bands found at lower wavenumbers when recorded in D_2_O. This is consistent with protection of the dppz ligand of both enantiomers from the aqueous solvent upon intercalation of the complex in the DNA decamer duplex. Interestingly the transient band attributed to the ancillary phen ligand (at 1518 cm^–1^) is significantly more intense than the dppz-based broad transient absorption at 1396 cm^–1^. This relative weakening of the intensity of the dppz bands upon intercalation is consistent with suppression of the ring vibrations of the dppz ligand upon π-stacking with the aromatic DNA bases, which is in accordance with previous IR studies.^69^ On the other hand the ancillary phen ligands of **1** are located in the DNA groove and therefore would not be expected to be dampened.^68^,^70^,^71^ No significant differences were observed between the TRIR spectra of the two enantiomers of **1** when bound to DNA. This suggests that the different orientation of the two enantiomers when intercalated into DNA, as indicated by solution studies and our recent crystal structure, do not strongly affect the TRIR spectrum of **1**.^4^–^6^,^70^,^72^ It is interesting to note that, based on the calculations above, in order for the dark state to form, the complex must be H-bonded to water molecules and be in an aqueous medium. However, while intercalated between the DNA base pairs the complex experiences an organic environment and therefore the dark state should not form, even if one binding mode leaves the nitrogens of the dppz exposed to water coordination. At higher wavenumbers (1600–1700 cm^–1^, shown within the green box in Fig. 8c), strong bleaching is noted in the TRIR spectra. In this region the DNA nucleobases absorb strongly but the complex does not (Fig. 8a and b).^51^,^73^ Two particularly strong bleach bands are observed at 1680 and 1648 cm^–1^, which correspond to the frequencies of the guanine and cytosine carbonyl stretches.^73^ Similar behaviour has also been seen in the [Ru(TAP)_2_(dppz)]^2+^ analogue (TAP = 1,4,5,8-tetraazaphenanthrene),^43^,^74^,^75^ and for a number of substituted dppz complexes of rhenium when intercalated into DNA.^50^,^76^ As DNA is not excited at 400 nm and **1** does not undergo efficient electron transfer with DNA in the picosecond time range,^77^ the presence of these bands is attributed to perturbation of the base pairs' vibrations at the site of intercalation of **1**. This perturbation possibly arises from an electrostatic (Stark) effect caused by the change in electron density of the metal complex upon photo-excitation.^78^,^79^ Of particular interest here is the strong difference of the signals in the DNA region of the TRIR spectrum induced by the enantiomers of **1** (racemic-**1** shows intermediate behaviour – see Fig. S13 in the ESI†). In particular the bleach of the guanine-localised band at 1680 cm^–1^ is much stronger for the Λ-complex than the Δ, which indicates that the guanine nucleotide is more perturbed by this optical enantiomer. It is expected that the enantiomers will show different preferences for binding to the various sites in the duplex DNA and that even if they do intercalate at the same site there may be a different orientation of the dppz group. Other studies suggest, for example, that the Λ-complexes have a preference for pyr·pur/pyr·pur or pur·pur/pyr·pyr steps.^71^,^74^ For these latter steps crystal structures and molecular modelling show that Λ-**1** is more strongly angled (canted) in the intercalation pocket,^71^,^80^ which affords greater overlap with the purine base (Fig. 9). Therefore binding of a significant fraction of the Λ-enantiomer at the G_3_G_4_/C_7_C_8_ step would be consistent with these results. However further studies with a range of sequences will be required to determine definitively the exact origin of this effect.

## Conclusions

The ps-TRIR spectra of **1** in CD_3_CN and D_2_O show strikingly different features, presenting characteristic vibrational marker bands allowing direct identification of both the emissive “bright” and non-emissive “dark” excited states in solution. The vibrations responsible for these bands were found to originate on the dppz ligand of the complex. DFT calculations suggest that these spectral features can only be accounted for by an explicit treatment which takes account of both solvent polarity and the inclusion of two D_2_O molecules H-bonded to the nitrogens of the phenazine moiety. Our results strongly suggest that the coupling of the modes of vibration of these H-bonded D_2_O molecules facilitate deactivation of the excited state by more efficient transfer of energy to the bulk water resulting in the extremely short lifetime of the excited state in aqueous media. The spectral features of the “bright” excited state were observed for both Δ- and Λ-enantiomers of **1** when bound to duplex DNA. Moreover, a very significant difference in the interaction of the complexes was observed by TRIR in the spectral region where the nucleobases absorb. This demonstrates that TRIR may prove to be a very valuable technique for determining the identity and nature of the binding site for molecules binding to mixed sequence duplex DNA. The TRIR method may also be useful for probing non-canonical structures of nucleic acids^53^,^81^,^82^ and for other biomolecules such as those found in Alzheimer plaques.^83^

## Experimental

### Materials and methods

Complexes **1**, **2**, and **3** were synthesised as their Cl and PF_6_ salts by modified literature procedures (see ESI† for details).^6^,^16^,^52^ All chemicals were obtained from Sigma-Aldrich, Alfa Aesar or TCI and unless specified, were used without further purification. Prior to use the Amberlite IRA-400 (chloride form) resin was soaked in methanol (HPLC) and washed thoroughly with fresh methanol and water. The resin was then subjected to one regeneration cycle where the beads were washed with NaOH (0.1 M) and then with water until the washings ran neutral. The beads were then washed with HCl (0.1 M) and water until the washings were neutral. Deuterated solvents for NMR use were purchased from Apollo Ltd. Analytical TLC was performed using Merck Kieselgel 60 F254 silica gel plates. Chromatographic columns were run using silica gel 60 (230–400 mesh ASTM). NMR spectra were recorded using an AV-700, AV-600 or DPX400 spectrometer, operating at 700, 600.1 or 400 MHz respectively for ^1^H NMR and 176 or 150.6 MHz respectively for ^13^C NMR. Shifts are referenced relative to the internal solvent signals. Mass spectra were recorded on a MALDI QToF Premier, running Mass Lynx NT V 3.4 on a Waters 600 controller connected to a 996 photodiode array detector with HPLC-grade methanol or acetonitrile. High resolution mass spectra were determined by a peak matching method, using glu-fib, as the standard reference (*m*/*z* = 1570.677). All accurate masses were reported within ±5 ppm. Infrared spectra were recorded on a Perkin Elmer Spectrum One FT-IR spectrometer fitted with a Universal ATR Sampling Accessory for solid samples or N_2_ flushed Transmission Accessory for liquid samples between CaF_2_ plates with an optical path length of 100 or 150 µm and at a resolution of 4 cm^–1^ and 256 scans.

Diffraction data for **1** was collected on a Bruker APEX 2 DUO CCD diffractometer using graphite-monochromatised Incoatec IµS MoKα (*λ* = 0.71073 Å) radiation. Crystals were mounted in a cryoloop/MiTeGen micromount and collected at 100(2) K using an Oxford Cryosystems Cobra low temperature device. Data were collected using omega and phi scans and were corrected for Lorentz and polarisation effects,^84^ and a multiscan absorption correction was carried out.^85^ The structure was solved using direct methods and refined by full-matrix least-squares procedures on *F*^2^ using SHELXL 2013 (ref. 86) within the OLEX2 software package.^87^ Hydrogen atoms were added geometrically in calculated positions and refined using a riding model. Slight crystallographic disorder was detected on the lattice acetonitrile molecule C502–C501–N501, which was modelled across two half-occupancy orientations with a PART-1 command to prevent incorrect connectivity, and an isotropic approximation restraint was used on the terminal methyl carbon atom C502 to maintain sensible anisotropic displacement parameters (see ESI†).

The oligonucleotide d(TCGGCGCCGA) was synthesised by ATDBio Ltd (Southampton, UK). The sequence was desalted and purified by gel filtration. An extinction coefficient of 163 000 M^–1^ cm^–1^ (duplex) at 260 nm was used for concentration determination.

### Transient spectroscopy methods

ps-TRIR measurements were performed on the ULTRA apparatus at the Central Laser Facility (STFC Rutherford Appleton Laboratories, Harwell, UK). The time-resolved IR (TRIR) spectrometer comprises of a 10 kHz repetition rate titanium sapphire dual output amplifier (Thales), producing 0.8 mJ output with 40 fs pulse duration, at 800 nm. Optical parametric amplifiers (Light Conversion, TOPAS) and second harmonic generation of the 800 nm beam created the mid-infrared radiation and 400 nm femtosecond pump pulses, respectively, used in these experiments. The polarisation of the pump pulses at the sample were at the magic angle relative to the probe, with an energy of 1 µJ. The IR probe beam was split to form reference and probe beams which were passed through spectrographs onto MCT array detectors (IR Associates). The 400 nm pump beam was mechanically chopped down to 5 kHz, focused (∼100 µm spot sizes) and overlapped with the probe beam (∼50 µm spot size) in the sample cell. High speed data acquisition systems (Quantum Detectors) allowed 10 kHz acquisition and processing of the probe and reference pulses to generate a pump-on pump-off infrared absorption difference signal. The temporal resolution in the ps-TRIR experiments is estimated to be about 150 fs. The TRIR spectra were calibrated using the characteristic polystyrene absorption lines.

Samples for ps-TRIR and FTIR were loaded into a demountable solution IR cell (Harrick Scientific Products Inc., New York) fitted with 25 mm diameter CaF_2_ windows (Crystran Ltd, UK), separated by Teflon spacers of 50 or 100 µm path length, as follows: (i) solutions of [**1**]·2Cl (500 µM), [**2**]·2Cl (300 µM) or [**3**]·2Cl (500 µM) in D_2_O (35 µL) were dropped between the CaF_2_ windows ([**2**]·2Cl was found to have limited solubility in D_2_O, saturation at *ca.* 440 µM, therefore its concentration was kept at 300 µM for experiments). (ii) Solutions of [**1**]·2PF_6_ (500 µM), [**2**]·2PF_6_ (500 µM) and [**3**]·2Cl (500 µM) in CD_3_CN (500 µL) were injected between two windows; (iii) solutions of Δ--**1**·2Cl or Λ-**1**·2Cl. (400 µM) in the presence of d(TCGGCGCCGA) (1 mM single stranded) in potassium phosphate buffer (50 mM, pH 7) in D_2_O were dropped between two CaF_2_ windows. Samples for ps-TRIR experiments were raster scanned in the *x* and *y* directions to minimise photodamage and re-excitation effects. All experiments were carried out at room temperature (*ca.* 19 °C). Samples were checked before and after the experiment by UV/vis spectroscopy to ensure no sample decomposition was occurring. To obtain the kinetics of a transient band or depletion, a subtraction of the ΔOD value at a nearby wavenumber showing no transient bands or depletions was performed from the wavenumber of interest. This was done in order to account for any baseline fluctuations during the experiment.

## Supplementary Material

Supplementary information

Supplementary information

Supplementary information

Supplementary information

Supplementary information

Crystal structure data
