# Supplementary figures and images for: Direct observation by time-resolved infrared spectroscopy of the bright and the dark excited states of the [Ru(phen)2(dppz)]2+ light-switch compound in solution and when bound to DNA
Source: Chem Sci. 2016 Jan 27;7(5):3075–84. doi: 10.1039/c5sc04514b (PMC6005197; doi:10.1039/c5sc04514b)

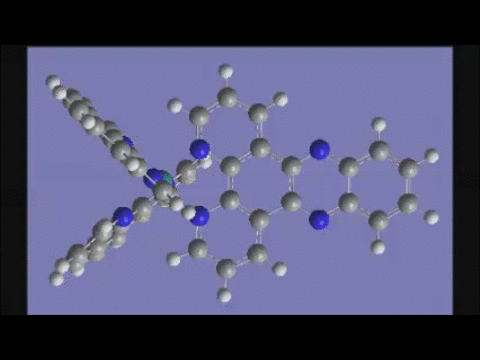

Supplement: Supplementary file 1 [file SC-007-C5SC04514B-s001.gif]

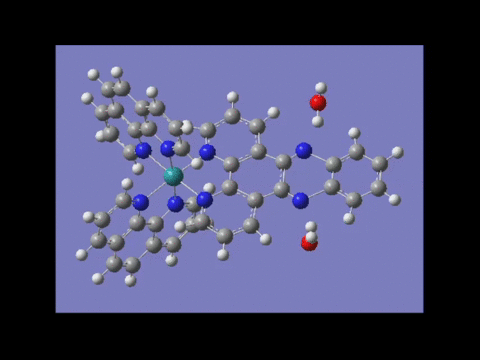

Supplement: Supplementary file 2 [file SC-007-C5SC04514B-s002.gif]

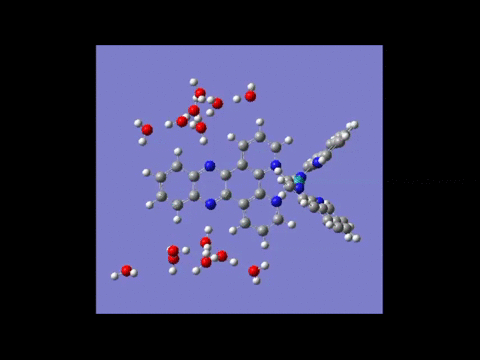

Supplement: Supplementary file 3 [file SC-007-C5SC04514B-s003.gif]

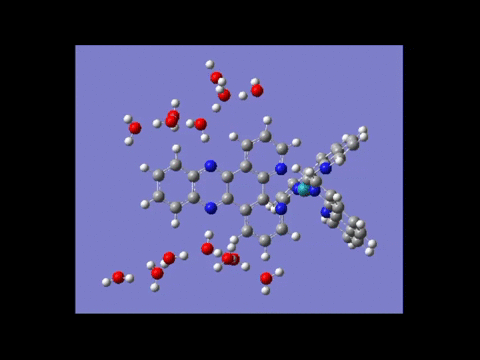

Supplement: Supplementary file 4 [file SC-007-C5SC04514B-s004.gif]
