# Supplementary material for: Direct observation by time-resolved infrared spectroscopy of the bright and the dark excited states of the [Ru(phen)2(dppz)]2+ light-switch compound in solution and when bound to DNA
Source: Chem Sci. 2016 Jan 27;7(5):3075–84. doi: 10.1039/c5sc04514b (PMC6005197; doi:10.1039/c5sc04514b)
Supplement: Supplementary file 5 [file SC-007-C5SC04514B-s005.pdf]

Electronic Supplementary Material (ESI) for Chemical Science.
This journal is © The Royal Society of Chemistry 2016

Supporting Information

Direct Observation by Time-resolved Infrared Spectroscopy of the Bright and the Dark Excited States of the $[Ru(phen)_2(dppz)]^{2+}$ Light-switch Compound in Solution and when Bound to DNA

Fergus E. Poynton,[a,b] James P. Hall,[c,d] Páraic. M. Keane,[a,c] Christine Schwarz,[c] Igor V. Sazanovich,[e] Michael Towrie,[e] Thorfinnur Gunnlaugsson,[a,b] Christine J. Cardin,[c] David J. Cardin,[c] Susan J. Quinn,[f] Conor Long,*[g] John M. Kelly*[a]

[a.] School of Chemistry, Trinity College Dublin, The University of Dublin, Dublin 2, Ireland.

[b.] Trinity Biomedical Sciences Institute (TBSI), Trinity College Dublin, The University of Dublin, Dublin 2, Ireland.

[c.] Department of Chemistry, University of Reading, Reading RG6 6AD, UK;

[d.] Diamond Light Source, Harwell Science and Innovation Campus; Didcot, Oxfordshire,OX11 0QX, UK.

[e.] Central Laser Facility, Research Complex at Harwell, STFC Rutherford Appleton Laboratory, Oxfordshire, OX11 0QX.

[f.] School of Chemistry, University College Dublin, Dublin 4, Ireland.

[g.] The School of Chemical Sciences, Dublin City University, Dublin 9, Ireland.

Email: jmkelly@tcd.ie, conor.long@dcu.ie

Contents

Nanosecond Transient Spectroscopy Methods 2
Synthesis of Λ & Δ $[Ru(phen)_2(phen\text{-}Dione)]2Cl$ 2
Synthesis of Complex [**2**]2Cl:[2, 3] 4
Crystal Structure of Complex 1 5
Spectroscopy of homoleptic complexes. 10
Ground State IR Bands: 11
Tables of TRIR Bands: 12
ns-TRIR spectra 14
Computational Method: 16
Computational Material 17
DNA Binding 21
References 22

Nanosecond Transient Spectroscopy Methods

For ns-TRIR experiments a sub-nanosecond laser (Picolo-AOT lasers from InnoLas Laser) with an output of 355 nm was used. The laser was externally triggered at 10 kHz and synchronized electronically to the ultrafast beams using a computer-controlled digital delay generator (Stanford DG645) to access nanosecond to microsecond time delays with approximately 100-ps precision. The laser beam was mechanically chopped before the sample at 5 kHz with a C-995 chopper from Terahertz Technologies Inc. The excitation pulse energy at the sample was ca. 0.8-1 μJ and focused into a ca. 100 μm spot. The polarization of the pump pulses at the sample was at the magic angle relative to the femtosecond probe beam. The probe light was generated by the same apparatus as described for the ps-TRIR.

Synthesis of Λ & Δ [Ru(phen)$_2$(phen-Dione)]2Cl

The bis(phenanthroline)(1,10-phenanthroline-5,6-dione)ruthenium complex as the dichloride was prepared from [Ru(phen)$_2$Cl$_2$] (0.56 g) and the dione (0.24 g) in a mixture of ethanol and water (1:1, 40 mL) under reflux for 3.5 h under a nitrogen atmosphere. The solvent was removed under vacuum, and the product dissolved in a small quantity of methanol (*ca.* 4 mL), the supernatant decanted, after which addition of *ca.* 10 mL of diethyl ether induced crystallisation over 10 hr at 4 ^{o}C, to give 0.58 g of brown crystals which were used without further purification. ^{1}H NMR (d_6-DMSO; 700 MHz, δ): 8.86, (1H, dd, J = 8.33, 1.05 Hz), 8.75 (1H, dd, J = 8.19, 0.98 Hz), 8.49 (1H, dd, J = 7.91, 1.33 Hz), 8.39, (2H, m), 8.28 (1H, d, J = 5.25 Hz), *ca.* 7.93 (2H, m), 7.88 (1H, dd, J = 5.67, 1.33 Hz), 7.72 (1H, m), 7.57 (1H, m); ^{13}C NMR (d_6-DMSO, 176 MHz, δ): 173.91, 156.06, 155.04, 153.00, 152.59, 147.02, 146.85, 137.24, 137.05, 134.71, 131.59, 130.49, 130.47, 128.42, 128.11, 128.05, 126.35, 126.20. The dione ruthenium complex was then resolved using 0.76 g dissolved in water (30 mL) heated almost to boiling. To this solution was added L(+)-sodium arsenyl tartrate (1.55 g, dissolved in 15 mL water), and crystallisation of the product initiated by scratching the vessel. On cooling to 4 ^{o}C (*ca.* 8 hr), the product crystallised out, was filtered off and washed with cold water, acetone, and ether. The crude product was purified by dissolution in hot

dimethylsulphoxide(12.5 mL) to which boiling water (40 mL) was added, followed by the Δ-sodium arsenyl tartrate enantiomer (0.425 g, dissolved in 5 mL hot water). Crystallisation was again initiated as previously, and the purification step repeated twice more. The crystals thus obtained were dissolved in hot acetic acid (50% aqueous, 8 mL), followed by water (8 mL), and a solution of ammonium hexafluorophosphate (2 mL containing 0.43 g). The product was filtered off, washed with cold water, ethanol, and ether, and dried under vacuum (0.22 g). ^{1}H NMR (d_6-DMSO, 700 MHz, δ) 8.86 (1H, dd, J = 8.35, 1.20 Hz), 8.75 (1H, dd, J = 7.10, 1.15 Hz), 8.51 (1H, dd, J = 6.65, 1.30 Hz), *ca.* 8.39 (2H, m), 8.28 (1H, dd, J = 4.05, 1.20 Hz), 7.99 (1H, d, J = 5.25 Hz), 7.97 (1H, dd, J = 3.00, 2.20 Hz), 7.84 (1H, dd, J = 4.30, 1.30 Hz), *ca.* 7.73 (1H, m), *ca.* 7.58 (1H, m); ^{13}C NMR (d_6-DMSO, 176 MHz, δ) 174.72, 156.64, 155.75, 153.43, 153.11, 147.60, 147.41, 137.78, 137,56, 135.30, 131.91, 131.03, 131.01, 128.96, 128.63, 128.55, 126.84, 126.65. For crystallisations the product was converted into the water-soluble chloride form using Amberlite anion exchange resin. ^{1}H NMR (CD_3CN, 400 MHz, δ): 9.67 (1H, dd, J = 8.24, 1.32 Hz), 8.66 (1H, dd, J = 4.52, 1.28 Hz), 8.51 (1H, dd, J = 6.60, 3.4 Hz), 8.30 (2H, s), 8.24 (1H, dd (J = 5.28, 1.32 Hz), 8.17 (1H m), 8.13 (1H, dd, J = 5.40, 1.32 Hz), 8.05 (1H, dd, J = 5.24, 1.24 Hz), 7.80 (1H, m), 7.08 (2H, m); ^{13}C NMR (CD_3CN, 176 MHz, δ): 154.38, 153.39, 153.12, 151.03, 148.04, 147.97, 142.93, 140.23, 137.11, 137.06, 133.60, 132.70, 131.23, 131.20, 130.92, 129.79, 128.235, 128.228, 127.36, 126.09, 126.04. The Δ-isomer was prepared using D(-)sodium arsenyl tartrate (in place of the L(+)-isomer; rather than recovering it from the liquor from the Λ-Ru dione resolution) and following the same procedure, except that the crude product was only recrystallized twice in total.

Subsequent reactions to synthesis Δ- and Λ-[**1**]2Cl were carried out according to a literature method.[1]

Synthesis of Complex [**2**]2Cl:[2, 3]

[Ru(dppz)$_2$Cl$_2$]·2H$_2$O (0.160 g, 0.20 mmol, 1 eq) and dipyrido[3,2-*a*:2',3'-*c*]phenazine (dppz) (0.062 g, 0.22 mmol, 1.1 eq) were suspended in EtOH:H$_2$O (1:1 ratio, 16 mL) and this solution was degassed by bubbling with argon for 20 mins. The mixture was then stirred and heated at 140 ˚C for 40 mins using microwave irradiation. The resulting solution was filtered and the PF_6 salt of the complex was precipitated from the filtrate by addition of a concentrated aqueous solution of NH_4PF_6. The resulting precipitate was isolated by centrifugation and washed with H_2O (5 mL x 2). The solid was dissolved in acetonitrile and purified by solid loading silica gel flash chromatography, eluting with CH_3CN/H_2O/aq. $NaNO_3$ (sat.) (40:4:1) (R_f = 0.46). The PF_6 salt of the complex was again precipitated and washed as described above. The resulting solid was further purified by precipitation through slow diffusion of diethyl ether into a solution of the complex in acetonitrile. The solid was isolated by filtration, washed with diethyl ether and dried under high vacuum to yield a red-brown solid (0.089 g, 36%).

The Cl complex was generated by swirling of the PF_6 complex in MeOH (20 mL) in the presence of Amberlite ion exchange resin (chloride form) for 40 mins. This suspension was filtered and the solvent removed under reduced pressure and the solid dried under high vacuum to yield a red-brown solid.

^{1}H NMR (600 MHz, CD_3CN, δ) 9.74 (dd, 6H, *J* = 8.2, 1.2 Hz), 8.55 – 8.50 (m, 6H), 8.35 (dd, 6H, *J* = 5.4, 1.2 Hz), 8.21 – 8.16 (m, 6H), 7.88 (dd, 6H, *J* = 8.2, 5.4 Hz). ^{13}C NMR (151 MHz, CD_3CN, δ) 155.5, 151.8, 143.8, 141.0, 134.8, 133.6, 132.0, 130.7, 128.4. ν_{max} (*ATR*)/cm^{-1}: 3662, 3080, 1980, 1618, 1601, 1579, 1545, 1510, 1492, 1464, 1451, 1429, 1413, 1359, 1341, 1306, 1224, 1206, 1189, 1148, 1118, 1097, 1080, 988, 876, 832, 810, 765, 737, 720. HRMS-MALDI$^+$ (*m/z*): [M]$^+$ calcd. for $C_{54}H_{30}N_{12}Ru$, 948.1760; found, 948.1800.

Crystal Structure of Complex 1

Table S1: Crystal data and structure refinement for complex **1**.

Identification code	**1**
Empirical formula	$2(C_{42}H_{26}N_8Ru)$, $4(PF_6)$, $5.5(C_2H_3N)$
Formula weight	2293.23
Temperature/K	100
Crystal system	monoclinic
Space group	$P2_1/c$
a/Å	20.7004(8)
b/Å	32.7257(12)
c/Å	14.3246(5)
α/°	90
β/°	107.6890(10)
γ/°	90
Volume/Å^3	9245.2(6)
Z	4
ρ_{calc}g/cm^3	1.648
μ/mm^{-1}	0.507
F(000)	4612.0
Crystal size/mm^3	0.28 × 0.131 × 0.064
Radiation	MoKα (λ = 0.71073)
2Θ range for data collection/°	3.234 to 54.992
Index ranges	$-26 \leq h \leq 26$, $-42 \leq k \leq 42$, $-18 \leq l \leq 18$
Reflections collected	436590
Independent reflections	21236 [R_{int} = 0.0602, R_{sigma} = 0.0261]
Data/restraints/parameters	21236/6/1339
Goodness-of-fit on F^2	1.080
Final R indexes [I>=2σ (I)]	R_1 = 0.0455, wR_2 = 0.0993
Final R indexes [all data]	R_1 = 0.0630, wR_2 = 0.1070
Largest diff. peak/hole / e Å^{-3}	1.12/-0.69

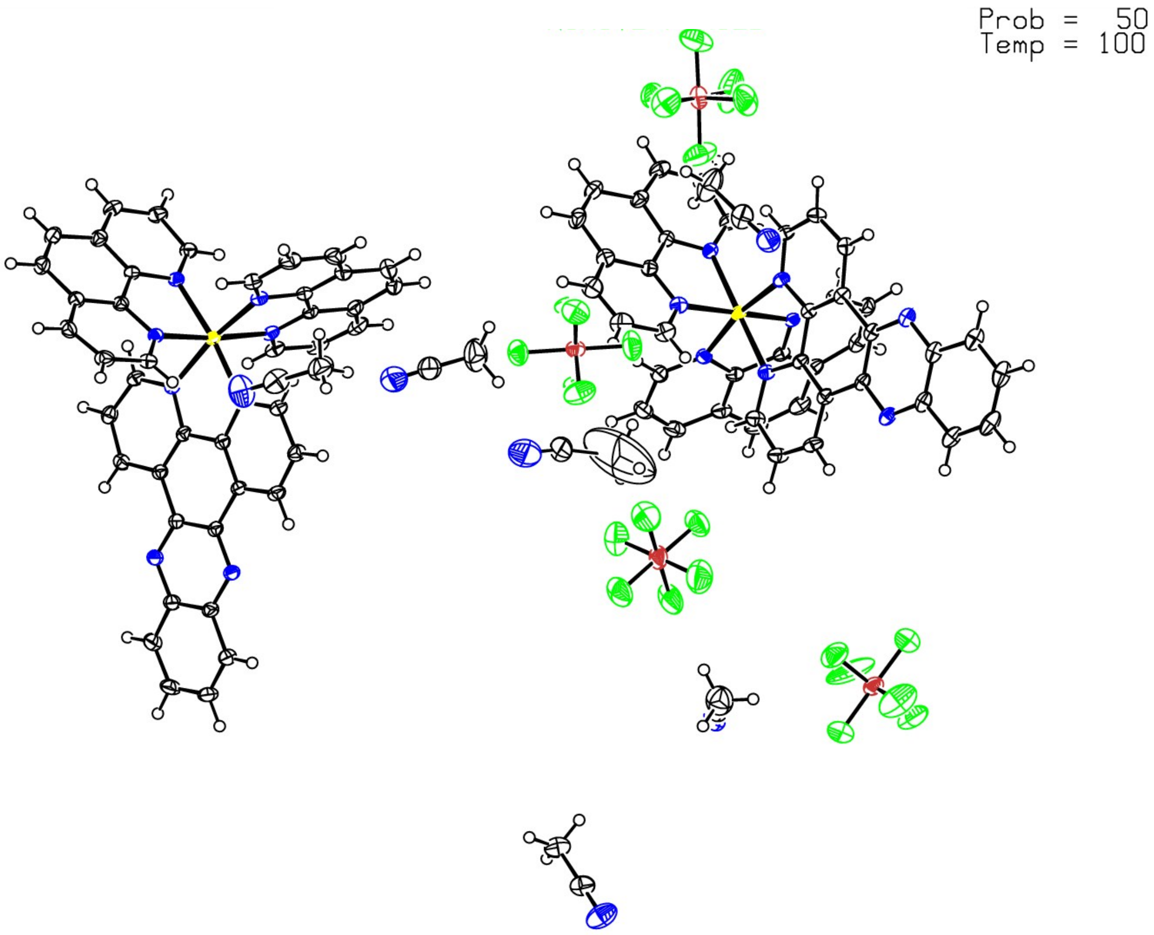

Fig. S1 The asymmetric unit of the X-ray crystal structure **1** containing the Δ and Λ enantiomers of **1**, four PF_6 counterions and six CH_3CN solvent molecules. Ellipsoids are shown at 50% probability.

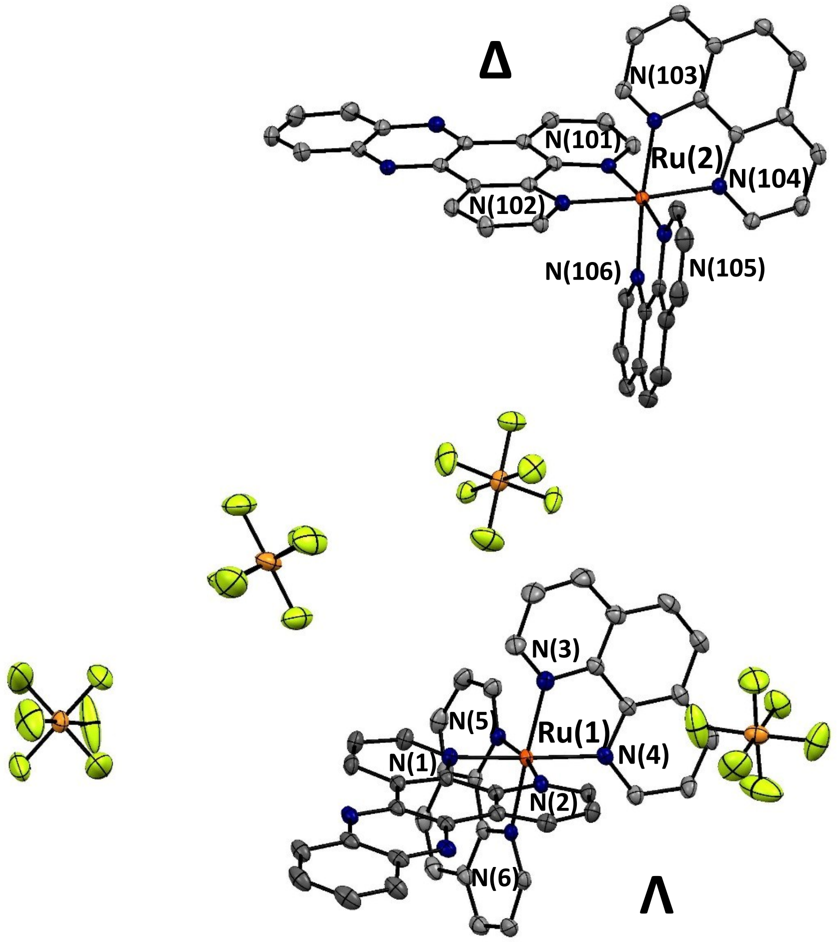

Fig. S2 The asymmetric unit of X-ray crystal structure of **1** showing the nitrogen numbering of the Δ and Λ enantiomers of **1** and four PF_6 counterions. Ellipsoids are shown at 50% probability.

Table S2: The bond lengths of the Ru-N coordination sphere in the crystal structure of Λ- and Δ-**1**.

Atom	Atom	Bond Length (Å)
**Ru(1)**	**N(1)**	2.065(2)
**Ru(1)**	**N(2)**	2.064(2)
**Ru(1)**	**N(3)**	2.061(3)
**Ru(1)**	**N(4)**	2.068(2)
**Ru(1)**	**N(5)**	2.060(2)
**Ru(1)**	**N(6)**	2.066(2)

Atom	Atom	Bond Length (Å)
**Ru(2)**	**N(101)**	2.078(2)
**Ru(2)**	**N(102)**	2.063(2)
**Ru(2)**	**N(103)**	2.069(2)
**Ru(2)**	**N(104)**	2.065(2)
**Ru(2)**	**N(105)**	2.058(3)
**Ru(2)**	**N(106)**	2.068(2)

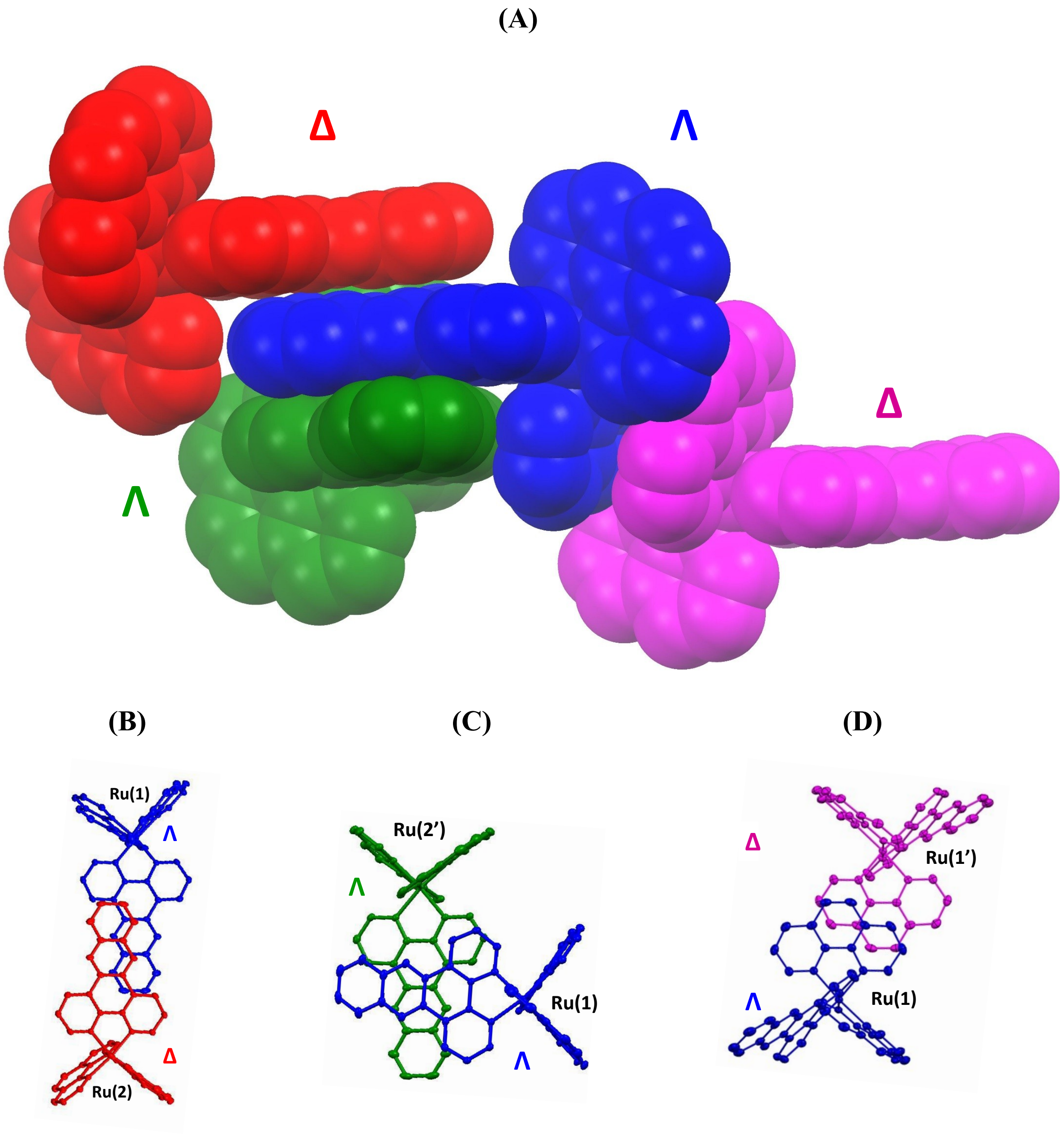

Fig. S3 A) Spacefill packing diagram showing the three π-stacking interactions undergone by each complex in the crystal of **1**: (B) head to tail π-stacking of the dppz ligands of opposite enantiomers (shown here for the Δ in red and the Λ in blue), (C) *ca.* 90˚ π-stacking of the dppz moieties of two of the same enantiomer (shown here for the two Λ enantiomers in blue and green) and (D) π-stacking of two ancillary phen ligands of a Λ and Δ complex (show here for the Λ enantiomers in blue and the Δ enantiomer in purple). Ru(1') and Ru(2') indicate that the complex is generated by an inversion centre and therefore corresponds to the opposite enantiomer.

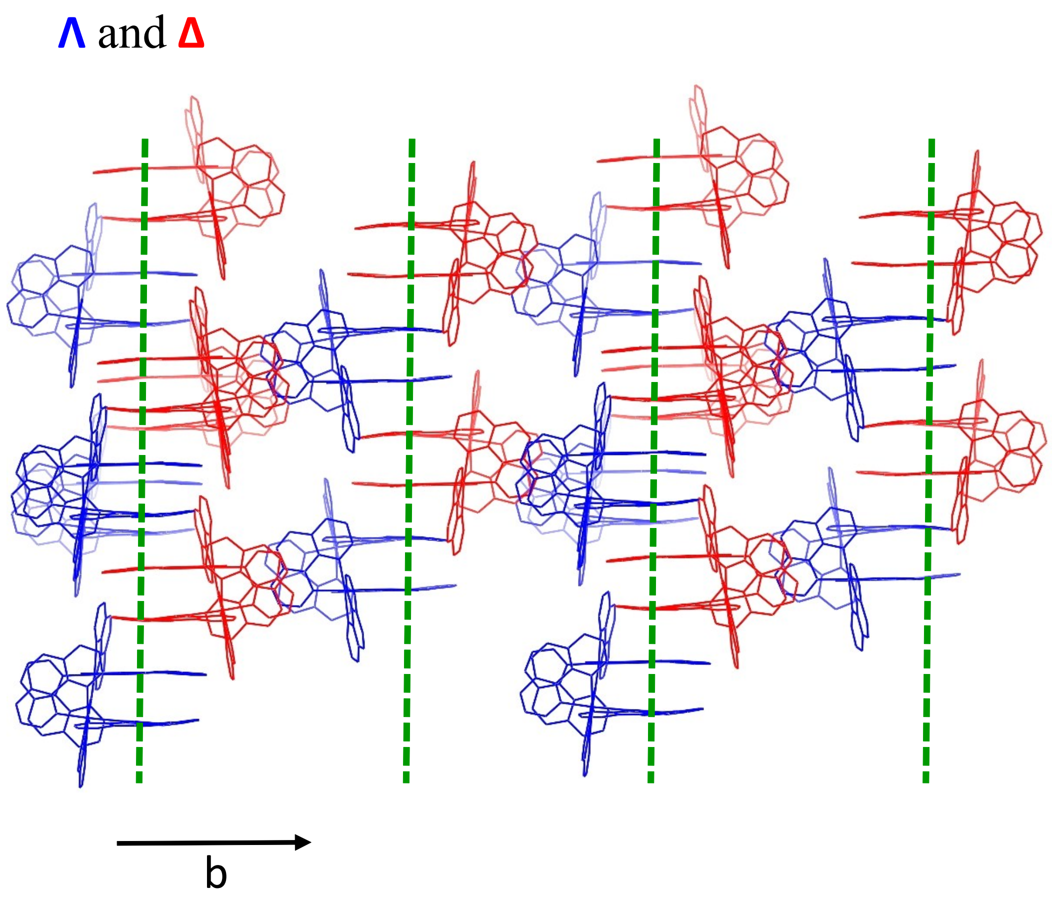

Fig. S4 Packing diagram showing the association of Λ (blue) and Δ (red) enantiomers of **1** in an AA-BB-AA fashion.

Spectroscopy of homoleptic complexes.

A) **B)**

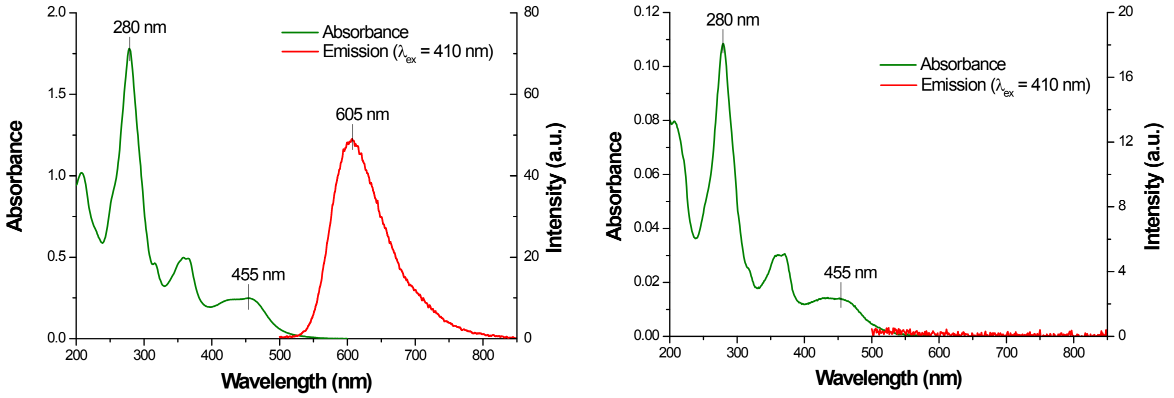

Fig. S5 UV-Vis absorption and emission spectra of **2** in A) CH_3CN (11.4 μM) and B) H_2O (2.3 μM), showing the solvent dependent luminescence of **2**.

A) **B)**

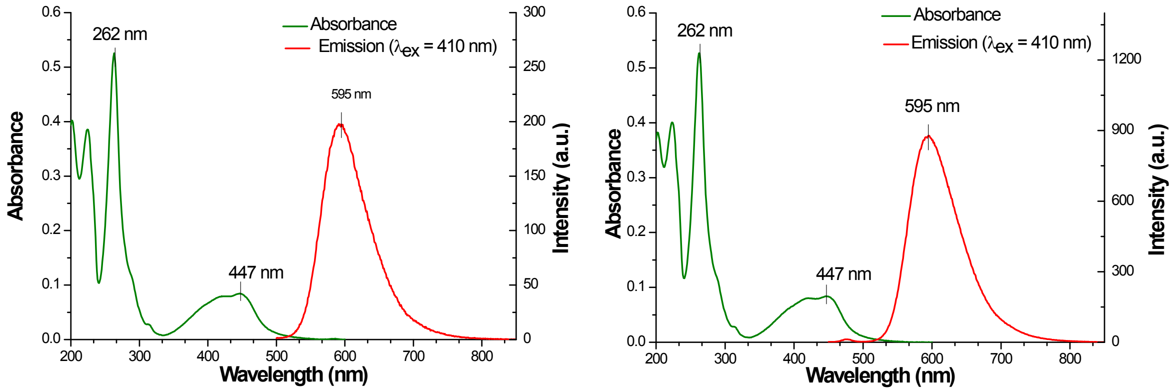

Fig. S6 UV-Vis absorption and emission spectra of **3** in A) CH_3CN (11.4 μM) and B) H_2O (2.3 μM).

Ground State IR Bands:

[**1**]2Cl 1 mM D_2O: ν_{max} (*Transmission*)/cm-1: 1307 (w), 1344 (m), 1361 (v. str), 1416 (m), 1431 (v. str), 1455 (w), 1465 (w), 1490 (m), 1552(w), 1582 (w), 1602 (w), 1633 (w).

[**2**]2Cl 0.4 mM D_2O: ν_{max} (*Transmission*)/cm^{-1}: 1311 (v. w), 1346 (w, sh), 1360 (v. str), 1423 (m), 1452 (w, sh), 1464 (m), 1495 (w), 1558 (m), 1598 (w), 1673 (str).

[**3**]2Cl 1 mM D_2O: ν_{max} (*Transmission*)/cm^{-1}: 1295 (w), 1343 (w), 1415 (str), 1430 (v. str), 1453 (w), 1493 (v. w), 1513 (v. w), 1543 (v. w), 1581 (w), 1602 (w), 1633 (w).

[**1**]2PF_6 1 mM CD_3CN: ν_{max} (*Transmission*)/cm^{-1}: 1342 (m), 1359 (v. str), 1422 (s), 1446 (w), 1466 (m), 1493 (s), 1549 (m), 1602 (w), 1630 (w).

[**2**]2PF_6 1 mM CD_3CN: ν_{max} (*Transmission*)/cm^{-1}: 1275 (v. w), 1308 (v. w), 1342 (m), 1359 (v. str), 1422 (s), 1447 (w), 1464 (m), 1493 (s), 1549 (s), 1602 (w), 1630 (w).

[**3**]2Cl 1 mM CD_3CN: ν_{max} (*Transmission*)/cm^{-1}: 1292 (v. w), 1310 (v. w), 1342 (w), 1413 (s), 1429 (v.s), 1453 (w), 1493 (w), 1513 (v. w), 1580 (w), 1602 (w), 1631 (w).

Tables of TRIR Bands:

Table S3: Table of transient vibrational bands of **1**, **2** and **3** in CD_3CN.

**[1)]2PF$_6$ CD$_3$CN**	**[2]2PF$_6$ CD$_3$CN**	**[3]2Cl CD$_3$CN**
-	-	1295 (bl ; w)
1313 (tr ; w)	1314 (tr; v. w)	-
1343 (bl ; w)	1341 (bl; v. w)	1342 (bl, v.w)
-	-	1346 (tr ; m)
1361 (bl ; str)	1359 (bl ; v. str)	-
1366 (tr ; sh)	1364 (tr ; w)	-
-	-	1390 (tr ; str)
1396 (tr ; v. str, (br))	1396 (tr ; st (br))	-
1414 (bl; w)	-	1411 (bl ; str)
1418 (tr ; w)	-	-
-	1422 (bl; m)	-
1430 (bl; m)	1430 (tr ; m)	1427 (bl ; v. str)
1435 (tr ; str)	-	1432 (tr; m)
-	1443 (bl; w)	
1458 (tr ; w)	1455 (tr ; m)	1455 (tr ; str)
-	1465 (bl; v. w)	-
1475 (tr ; m)	1475 (tr ; m)	-
1492 (bl ; w)	1493 (bl; v. w)	1491 (bl; w)
1498 (tr ; v. w (sh))	1500 (tr ; str)	-
-	1510 (tr ; m (sh))	-
1516 (tr ; v. str)	-	1518 (tr ; v. str)
1547 (bl; w)	1544 (bl ; m)	-
-	1552 (tr ; w)	-
1558 (tr ; v. w)	-	1560 (tr ; m)
-	1571 (tr. w)	-
1582 (tr ; v. w)	-	1581 (tr ; v. w)
-	1599 (tr ; w)	1601 (tr ; w)
1605 (tr ; v. w)	-	-
1634 (tr ; v. w)	-	1630 (tr ; v. w)

Table S4: Table of transient vibrational bands of **1**, **2** and **3** in D_2O.

**[1]2Cl D_2O**	**[2]2Cl D_2O**	**[3]2Cl D_2O**
-	1275 (bl; w)	-
1290 (tr; v. str)	1288 (tr ; str)	-
1321 (tr; v. str)	1315 (tr ; str)	-
-	-	1343 (bl; v. w)
1345 (tr; v. str)	1345 (tr ; str)	1348 (tr ; w)
1361 (bl; m)	1361 (bl; str)	-
1396 (tr; w & br)	1396 (tr ; m & br)	1392 (tr ; m)
-	-	1413 (bl; m)
1429 (tr; str)	1429 (tr ; str)	1429 (bl; str)
1435 (tr; v. str)	-	1436 (tr ; w)
-	1448 (bl; w)	-
-	1455 (tr; m)	-
1460 (tr; m)	1465 (bl; m)	1460 (tr ; m & br)
1480 (tr; m)	1480 (tr ; m)	-
-	1495 (bl, m)	1491 (bl; v. w)
1505 (tr; m)	1500 (tr ; str)	-
1518 (tr; v. str)	-	1518 (tr ; v. str)
-	1539 (tr ; w)	-
1544 (tr; m)	1547 (bl; w)	-
1560 (tr; m)	1555 (tr ; m)	1560 (tr ; w)
1585 (tr; m)	-	1581 (tr ; v. w)
1602 (tr; w)	1597 (bl; w)	1600 (tr ; w)
1634 (tr; w)	-	-

ns-TRIR spectra

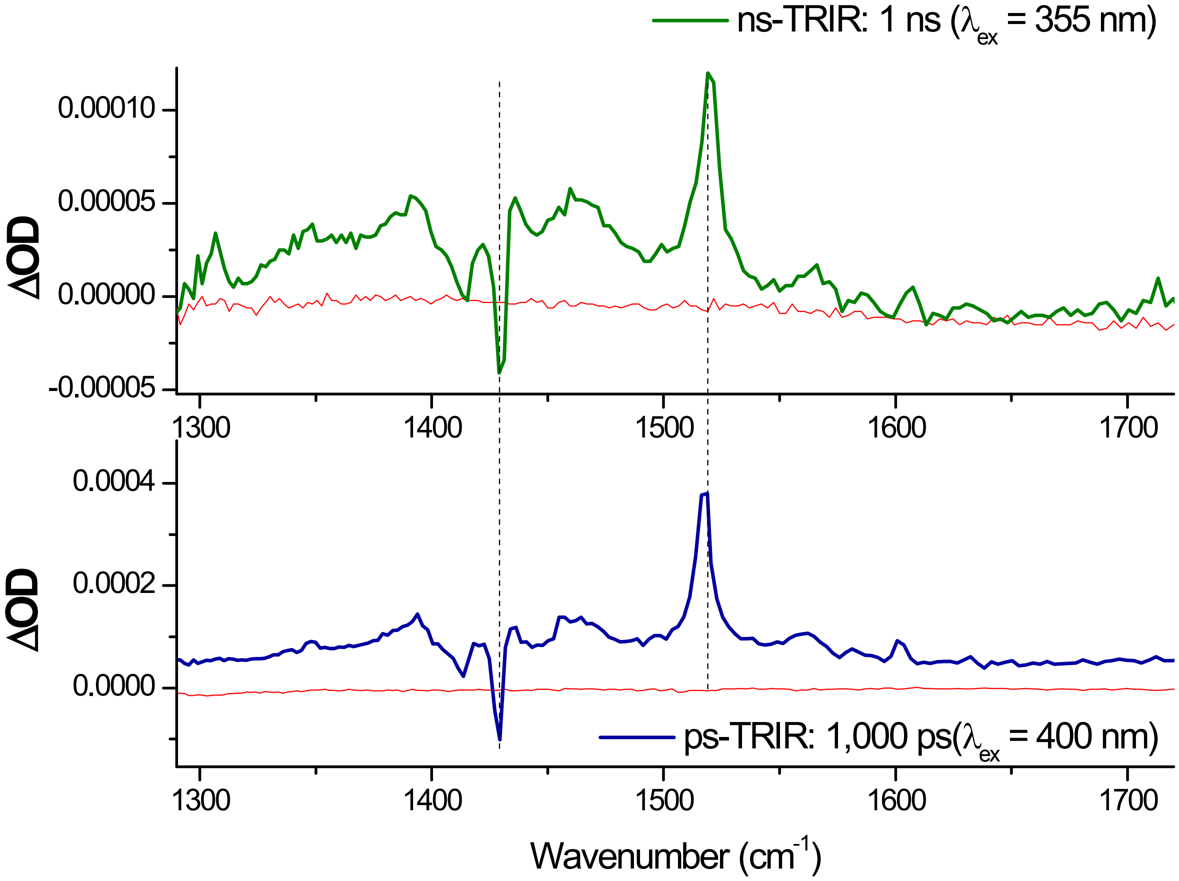

Fig. S7 TRIR spectra of [**3**]2Cl (500 μM) in D_2O at 1 ns after excitation at 355 nm (top) and 1,000 ps after excitation at 400 nm (bottom).

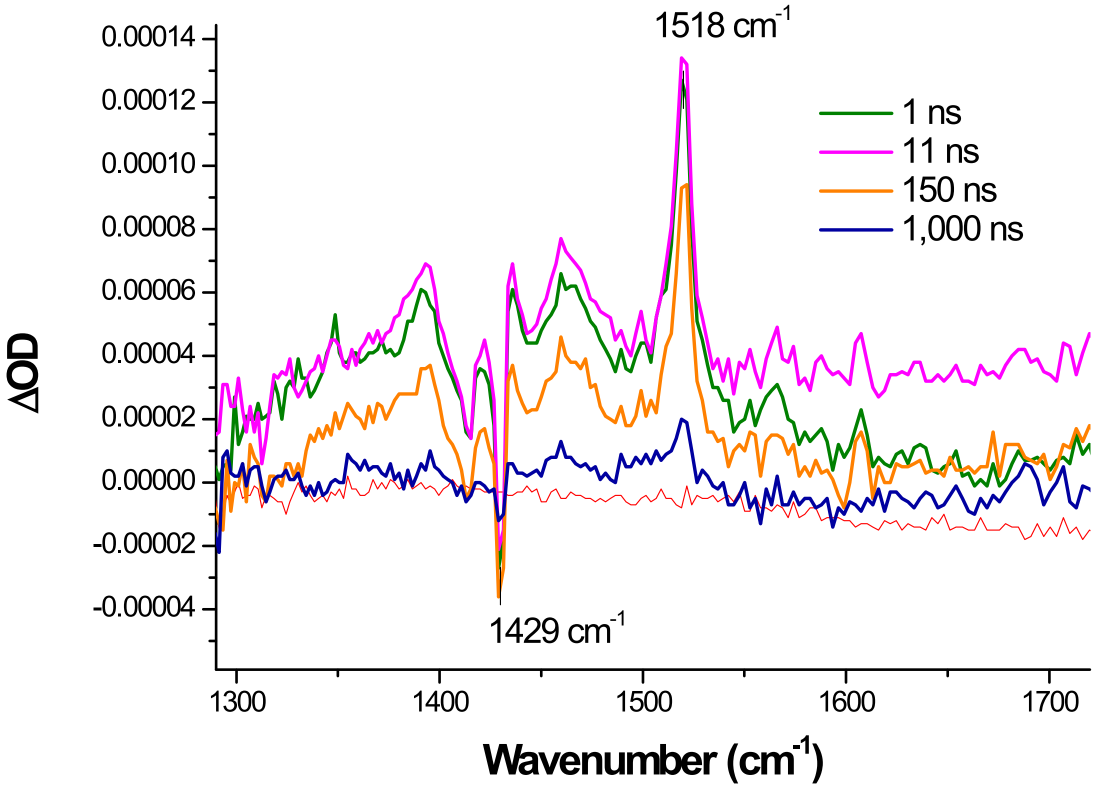

Fig. S8: ns-TRIR spectra of [**3**]2Cl (500 µM) in D_2O at various time delays after excitation of the complex at 355 nm.

Computational Method:

Density maps were visualised using GaussView 3.0. Corrections for solvent were applied using the polarisable continuum model,[4] water being used as the solvent for the hydrogen bonded complex while acetonitrile was used for the non-hydrated complex. In all cases the structural parameters were optimised on the triplet hypersurface. The nature of the lowest energy triplet state was then determined by subtracting the electron density map of the lowest energy singlet species at the triplet geometry from the electron density map of the triplet state. This allows a comparison on the electronic distribution in the lowest energy triplet state with that of the singlet ground state (Fig. S9) These calculations indicate that the nature of the lowest energy triplet state is affected by both the solvent medium and also the presence of hydrogen bonded water molecules. When the solvent is anhydrous acetonitrile, the lowest energy triplet state shows enhanced electron density on the A, B, C rings of the dppz, consistent with its assignment as a dppz(phen) MLCT state. In contrast when coordinated to two D_2O molecules (and in a water medium) the triplet state shows a greater involvement of the phenazine part (i.e. rings B, D, E) of the dppz ligand with a greater phenazine to metal charge transfer character. Examination of the spin density maps of the lowest energy triplet state reveals a similar result (see Fig. S10). Computational modeling of charge-transfer excited states, particularly those involving heavy metals, still poses a significant challenge. Various methodologies have been utilized in this endeavor, however a consensus on the most appropriate methodology to employ has yet to be definitively established in the literature.[5-8]

For FTIR simulations the experimentally observed bleach band at 1396 cm^{-1} was used for wavenumber calibration. This resulted in a correction factor of 0.977 being applied to the calculated wavenumbers. An error of 1% in the calculated positions of the IR bands corresponds to 15 cm^{-1}.

Computational Material

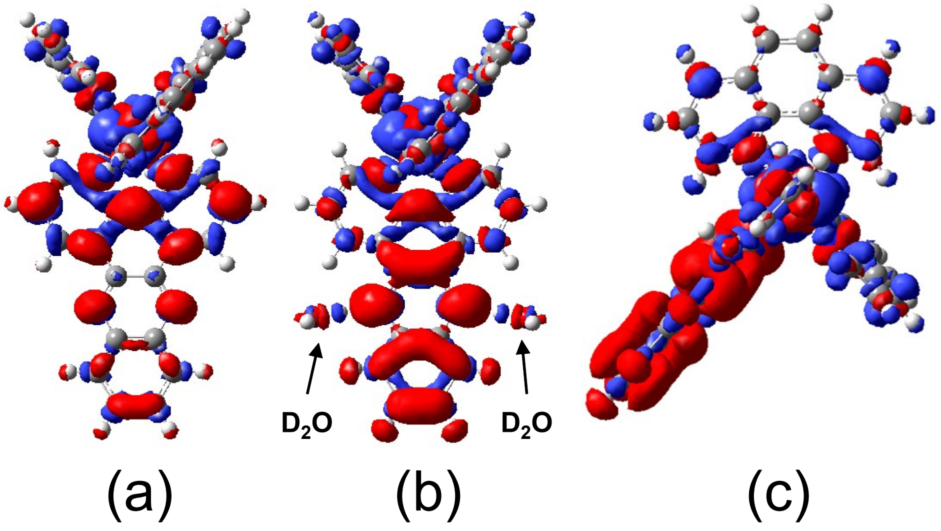

Fig. S9 (a) Electron density difference map (iso level 0.0008) between the lowest energy triplet state of **1** minus the electron density of the equivalent singlet ground-state at the same geometry in acetonitrile. (b) equivalent map for **1**·2(D_2O) in water; (c) the same map rotated to show the change in electron density on one of the phenanthroline ligands. The red volumes indicate the regions where the electron density is higher in the triplet state compared to the singlet and the blue volumes are where the electron density is less in the triplet compared to the singlet.

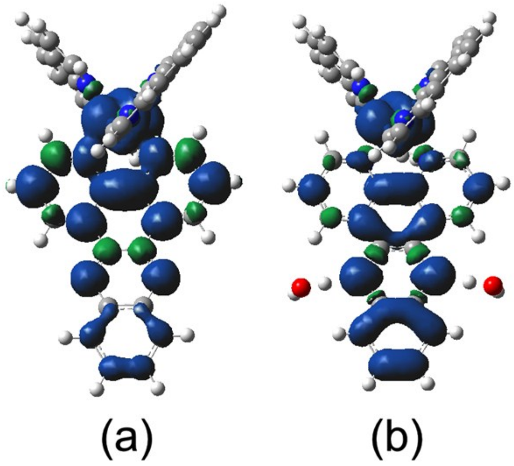

Fig. S10 Spin density maps of the lowest energy triplet state of a) **1** in acetonitrile and b) **1**·2(D_2O) in water.

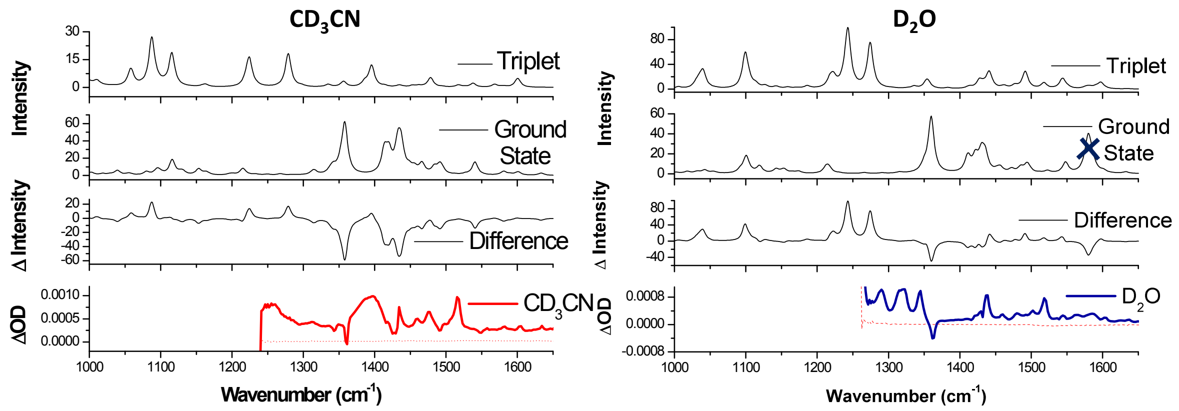

Fig. S11 The simulated infrared spectra of the ground state and lowest triplet states and their simulated difference spectra compared with the experimental time-resolved difference spectrum of **1** in acetonitrile (left column); and **1**·2(D_2O) in water (right column). Cross indicates that this calculated band at 1580 cm-1 in D_2O is composed of a number of O-D-O bending modes. In these calculations the bulk water was not modelled and therefore differences between the calculated and experimental O-D vibrations are to be expected.

Table S5: Selected bond lengths (Å) and angles (˚) for **1** (experimental and calculated) and **1**·$2H_2O$ (calculated) showing the mean unsigned deviation of the calculated values from those derived from the single crystal X-ray diffraction method for both the lowest energy singlet and triplet species.

			$Ru(phen)_2(dppz)$		$Ru(phen)_2(dppz)$		$Ru(phen)_2(dppz)2H_2O$	
			**Solvent**		**Solvent**		**Solvent**	
	**exp**	**exp**	**Acetonitrile**		**Water**		**Water**	
**Lengths**	**with esd**	**numeric**	**Singlet**	**Triplet**	**Singlet**	**Triplet**	**Singlet**	**Triplet**
Ru-N1	2.065(2)	2.065	2.09913	2.07911	2.09887	2.08121	2.09513	2.08850
Ru-N2	2.064(2)	2.064	2.09913	2.07911	2.09887	2.08121	2.09509	2.08900
Ru-N3	2.061(3)	2.061	2.10351	2.10164	2.10347	2.10159	2.10369	2.10432
Ru-N4	2.068(2)	2.068	2.10248	2.12176	2.10257	2.12054	2.10340	2.11274
Ru-N5	2.060(2)	2.06	2.10248	2.12176	2.10257	2.12054	2.10372	2.11262
Ru-N6	2.066(2)	2.066	2.10351	2.10164	2.10347	2.10159	2.10319	2.10371
**Angles**								
N1RuN2	79.69(9)	79.69	79.20181	80.76779	79.19958	80.69459	78.87286	79.72808
N1RuN4	174.85(9)	174.85	173.55770	175.08057	173.55165	175.14521	173.57047	175.3271
N2RuN5	169.72(9)	169.72	173.55777	175.08050	173.55149	175.14513	173.61877	175.35564
N3RuN4	80.07(10)	80.07	79.54930	79.41085	79.54424	79.42133	79.49351	79.47396
N3RuN6	171.98(10)	171.98	173.16110	176.02118	173.15694	175.97054	173.41171	175.84424
N5RuN6	80.04(10)	80.04	79.54926	79.41086	79.54428	79.42130	79.50262	79.48619

				Ru(phen)$_2$(dppz)		Ru(phen)$_2$(dppz)		Ru(phen)$_2$(dppz)2H$_2$O	
				Solvent		Solvent		Solvent	
	exp	exp		Acetonitrile		Water		Water	
**Phenazine lengths**	**with esd**	**numeric**		**Singlet**	**Triplet**	**Singlet**	**Triplet**	**Singlet**	**Triplet**
C7-C8	1.364(5)	1.364		1.3875	1.3952	1.3876	1.3964	1.3871	1.4018
C8-C9	1.407(5)	1.407		1.4346	1.4263	1.4346	1.4251	1.4333	1.4175
C9-C10	1.373(5)	1.373		1.3875	1.3952	1.3876	1.3964	1.3871	1.4018
C11-N8	1.357(4)	1.357		1.4280	1.3825	1.4280	1.4223	1.3724	1.3964
N7-C6	1.350(4)	1.35		1.3706	1.3825	1.3707	1.3841	1.3724	1.3963
C5-N7	1.334(4)	1.334		1.3455	1.3489	1.3455	1.3503	1.3465	1.3676
N8-C12	1.330(4)	1.33		1.3455	1.3489	1.3455	1.3503	1.3464	1.3677
			**Basis Set**	**LanL2DZ**	**LanL2DZ**	**LanL2DZ**	**LanL2DZ**	**LanL2DZ**	**LanL2DZ**
			**Functional**	**B3LYP**	**UB3LYP**	**B3LYP**	**UB3LYP**	**B3LYP**	**UB3LYP**
**Mean Unsigned Deviation**			**bonds**	0.0316	0.0297	0.0316	0.0333	0.0270	0.0355
			**angles**	1.3018	1.9997	1.3031	1.9971	1.4235	1.8608

The table values above suggest that the model chemistry adequately describes the complex in this study.

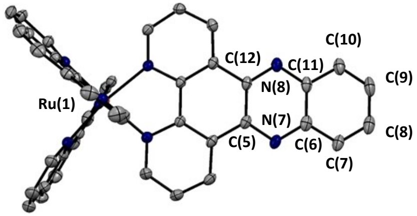

Fig. S12 The molecular structure of Λ-**1** showing relevant atom numbering from table S5. Ellipsoids shown at 50% probability.

DNA Binding

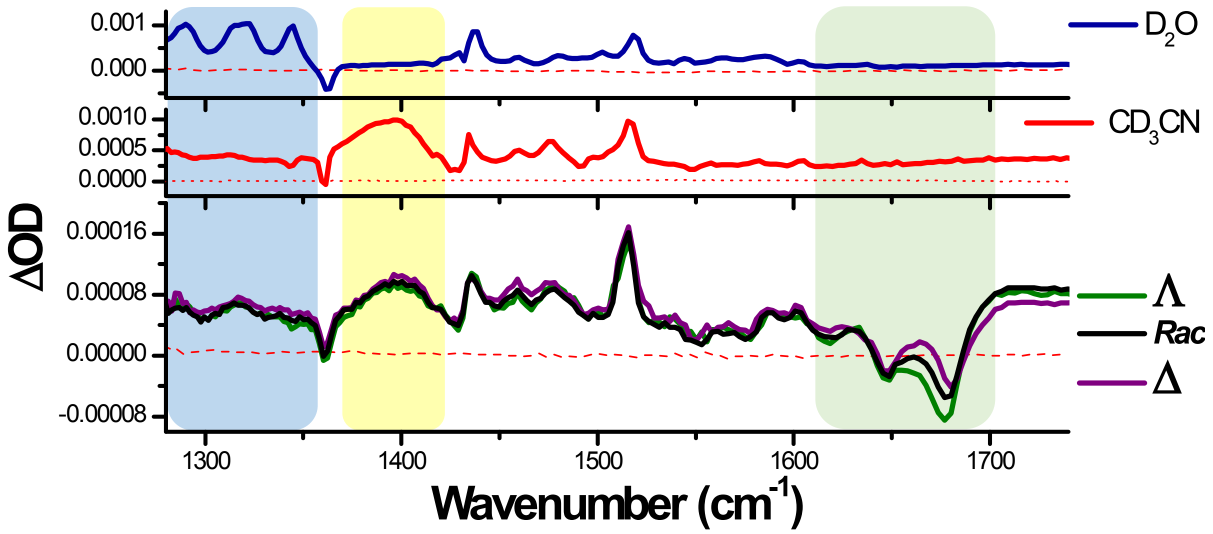

Fig. S13 TRIR spectra recorded 35 ps after 400 nm excitation of of a) **1**·2Cl (500 μM) in D_2O, b) **1**·$2PF_6$ (500 μM) in CD_3CN and c) Δ- and Λ- and rac-[**1**]2Cl (400 μM) and a duplex DNA oligonucleotide $d(TCGGCGCCGA)_2$ (500 μM duplex) in deuterated potassium phosphate buffer (50 mM) pH 7. The blue and yellow coloured regions highlight the characteristic transient bands for the excited state of **1**, while the green coloured region highlights the bands associated with the base DNA base pairs.

References

Full Gaussian reference:

Frisch, J.; Trucks, G. W.; Schlegel, H. B.; Scuseria, G. E.; Robb, M. A.; Cheeseman, J. R.; Scalmani, G.; Barone, V.; Mennucci, B.; Petersson, G. A.; Nakatsuji, H.; Caricato, M.; Li, X.; Hratchian, H. P.; Izmaylov, A. F.; Bloino, J.; Zheng, G.; Sonnenberg, J. L.; Hada, M.; Ehara, M.; Toyota, K.; Fukuda, R.; Hasegawa, J.; Ishida, M.; Nakajima, T.; Honda, Y.; Kitao, O.; Nakai, H.; Vreven, T.; Montgomery, J. A.; Peralta, J. E.; Ogliaro, F.; Bearpark, M.; Heyd, J. J.; Brothers, E.; Kudin, K. N.; Staroverov, V. N.; Kobayashi, R.; Normand, J.; Raghavachari, K.; Rendell, A.; Burant, J. C.; Iyengar, S. S.; Tomasi, J.; Cossi, M.; Rega, N.; Millam, J. M.; Klene, M.; Knox, J. E.; Cross, J. B.; Bakken, V.; Adamo, C.; Jaramillo, J.; Gomperts, R.; Stratmann, R. E.; Yazyev, O.; Austin, A. J.; Cammi, R.; Pomelli, C.; Ochterski, J. W.; Martin, R. L.; Morokuma, K.; Zakrzewski, V. G.; Voth, G. A.; Salvador, P.; Dannenberg, J. J.; Dapprich, S.; Daniels, A. P.; Farkas, O.; Foresman, J. B.; Ortiz, J. V.; Cioslowski, J.; Fox, D. J. *Gaussian 09*, Gaussian, Inc.: Wallingford CT, 2009.

1. C. Hiort, P. Lincoln and B. Norden, *J. Am. Chem. Soc.*, 1993, **115**, 3448-3454.
2. S. Rau, B. Schäfer, A. Grüßing, S. Schebesta, K. Lamm, J. Vieth, H. Görls, D. Walther, M. Rudolph, U. W. Grummt and E. Birkner, *Inorg. Chim. Acta*, 2004, **357**, 4496-4503.
3. S. M. Cloonan, R. B. P. Elmes, M. Erby, S. A. Bright, F. E. Poynton, D. E. Nolan, S. J. Quinn, T. Gunnlaugsson and D. C. Williams, *J. Med. Chem.*, 2015, **58**, 4494-4505.
4. J. Tomasi, B. Mennucci and R. Cammi, *Chem Rev*, 2005, **105**, 2999-3093.
5. A. Dreuw, J. L. Weisman and M. Head-Gordon, *J. Chem. Phys.*, 2003, **119**, 2943-2946.
6. R. J. Magyar and S. Tretiak, *J. Chem. Theory Comput.*, 2007, **3**, 976-987.
7. D. J. Tozer, *J. Chem. Phys.*, 2003, **119**, 12697-12699.
8. L. González, D. Escudero and L. Serrano-Andrés, *ChemPhysChem*, 2012, **13**, 28-51.
